# Crystal structure and proton conductivity of BaSn_0.6_Sc_0.4_O_3–*δ*_: insights from neutron powder diffraction and solid-state NMR spectroscopy†
†Electronic supplementary information (ESI) available: Rietveld fit of dry BaSn_0.6_Sc_0.4_O_3–*δ*_ sample (Fig. S1). ^119^Sn (Fig. S2), ^45^Sc (Fig. S3–S6) and ^17^O (Fig. S7) spectra of all materials as a function of Sc doping concentration, ^45^Sc MQMAS of deuterated BaSn_0.9_Sc_0.1_O_3–*δ*_ (Fig. S4), ^45^Sc MQMAS of dry and deuterated BaSn_0.8_Sc_0.2_O_3–*δ*_ (Fig. S5), ^45^Sc MQMAS of dry and deuterated BaSn_0.7_Sc_0.3_O_3–*δ*_ (Fig. S6), ^17^O MQMAS of ^17^O enriched BaSn_0.8_Sc_0.2_O_3–*δ*_ and BaSn_0.6_Sc_0.4_O_3–*δ*_ (Fig. S8). See DOI: 10.1039/c5ta09744d



**DOI:** 10.1039/c5ta09744d

**Published:** 2016-03-16

**Authors:** Francis G. Kinyanjui, Stefan T. Norberg, Christopher S. Knee, Istaq Ahmed, Stephen Hull, Lucienne Buannic, Ivan Hung, Zhehong Gan, Frédéric Blanc, Clare P. Grey, Sten G. Eriksson

**Affiliations:** a Department of Chemical and Biological Engineering , Chalmers University of Technology , SE-412 96 Gothenburg , Sweden; b The ISIS Facility , STFC Rutherford Appleton Laboratory , Didcot , Oxfordshire OX11 0QX , UK; c Department of Chemistry , State University of New York , Stony Brook , NY 11790-3400 , USA; d Center of Interdisciplinary Magnetic Resonance , National High Magnetic Field Laboratory , 1800 East Paul Dirac Drive , Tallahassee , Florida 32310 , USA; e Department of Chemistry , University of Cambridge , Lensfield Road , Cambridge , CB2 1EW , UK; f Department of Chemistry and Stephenson Institute for Renewable Energy , University of Liverpool , Crown Street , Liverpool , L69 7ZD , UK

## Abstract

The solid-state synthesis and structural characterisation of perovskite BaSn_1–*x*_Sc_*x*_O_3–*δ*_ (*x* = 0.0, 0.1, 0.2, 0.3, 0.4) and its corresponding hydrated ceramics are reported. Powder and neutron X-ray diffractions reveal the presence of cubic perovskites (space group *Pm*3*m*) with an increasing cell parameter as a function of scandium concentration along with some indication of phase segregation. ^119^Sn and ^45^Sc solid-state NMR spectroscopy data highlight the existence of oxygen vacancies in the dry materials, and their filling upon hydrothermal treatment with D_2_O. It also indicates that the Sn^4+^ and Sc^3+^ local distribution at the B-site of the perovskite is inhomogeneous and suggests that the oxygen vacancies are located in the scandium dopant coordination shell at low concentrations (*x* ≤ 0.2) and in the tin coordination shell at high concentrations (*x* ≥ 0.3). ^17^O NMR spectra on ^17^O enriched BaSn_1–*x*_Sc_*x*_O_3–*δ*_ materials show the existence of Sn–O–Sn, Sn–O–Sc and Sc–O–Sc bridging oxygen environments. A further room temperature neutron powder diffraction study on deuterated BaSn_0.6_Sc_0.4_O_3–*δ*_ refines the deuteron position at the 24k crystallographic site (*x*, *y*, 0) with *x* = 0.579(3) and *y* = 0.217(3) which leads to an O–D bond distance of 0.96(1) Å and suggests tilting of the proton towards the next nearest oxygen. Proton conduction was found to dominate in wet argon below 700 °C with total conductivity values in the range 1.8 × 10^–4^ to 1.1 × 10^–3^ S cm^–1^ between 300 and 600 °C. Electron holes govern the conduction process in dry oxidizing conditions, whilst in wet oxygen they compete with protonic defects leading to a wide mixed conduction region in the 200 to 600 °C temperature region, and a suppression of the conductivity at higher temperature.

## Abstract 2

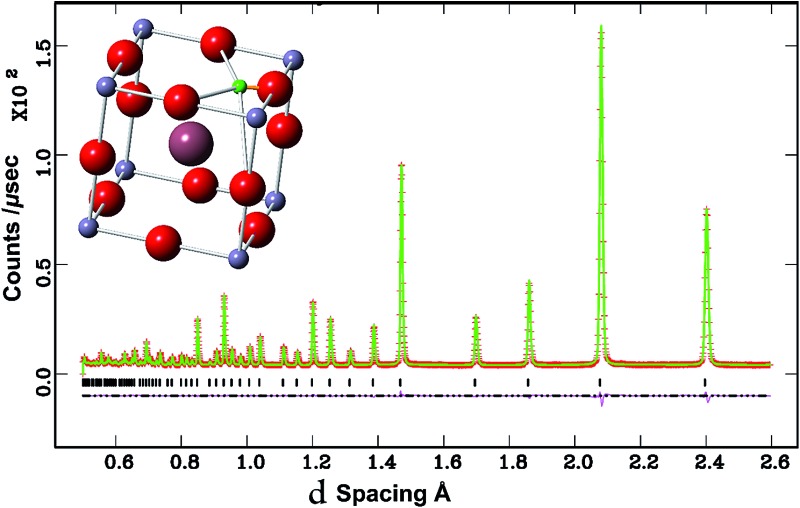
BaSn_0.6_Sc_0.4_O_3–*δ*_: location of the proton using neutrons with insights into its high conductivity and local environment using EIS and NMR.

## 1. Introduction

During the past three decades proton conducting ceramics have been widely studied due to their high ionic conductivities in the intermediate temperature region of 300–600 °C. In particular these materials have been proposed to be used as proton conducting electrolytes in protonic ceramic fuel cells (PCFC). The lower operating temperature of PCFCs would provide substantial advantages over solid oxide fuel cells (SOFC) based on oxide ion-conducting electrolytes. The higher operating temperatures of SOFCs, usually 700–900 °C, have limited their technological development due to high system costs, performance degradation rates, slow start-up and shutdown cycles. For example, in the 700–900 °C temperature range, the use of chromium containing interconnector steels might cause chromium poisoning of the electrodes^1^ and shorten the lifetime of the cell. Reduced start-up times and relaxed matching of the thermal expansion coefficients of the various fuel cell components are additional benefits that accompany the lowering of the operating temperature.

Acceptor doped perovskites provide many eligible systems for proton conducting electrolytes, *e.g.* BaZr_1–*x*_Y_*x*_O_3–*δ*_,^2–4^ BaCe_1–*x*_Y_*x*_O_3–*δ*_,^2^ SrCe_1–*x*_Y_*x*_O_3–*δ*_,^5^ BaZr_1–*x*_Yb_*x*_O_3–*δ*_ (ref. 6) all with 0 ≤ *x* ≤ 0.2, and BaZr_1–*x*_In_*x*_O_3–*δ*_ (ref. 7) with 0 ≤ *x* ≤ 1. Below 700 °C, BaZr_1–*x*_Y_*x*_O_3–*δ*_ possesses a bulk proton conductivity greater than the best oxide ion conductors.^2^ Proton incorporation is reliant on the formation of oxygen vacancies in a process commonly referred to as acceptor doping in the A^2+^B^4+^O_3_^2–^ type perovskites. Here, a portion of the tetravalent cations at the B-site is substituted by trivalent dopant cations resulting in the formation of charge compensating oxygen vacancies in the system. This process can be described using Kröger–Vink notation for Sc-doped BaSnO_3_ with Sc^3+^ doped on the Sn^4+^ site as:1
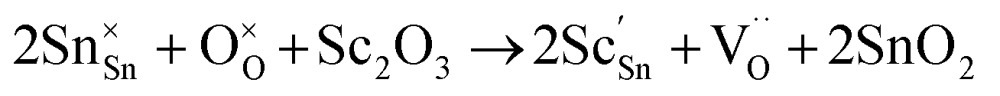
with 
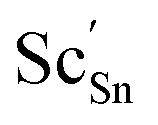
 corresponding to a Sc^3+^ ion sitting on a Sn site with a negative charge, and 
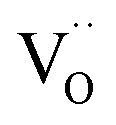
 to an oxygen vacancy with two positive charges. When in contact with a H_2_O bearing gas the oxygen vacancies 
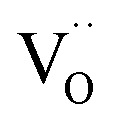
 are filled *via* the following reaction:2
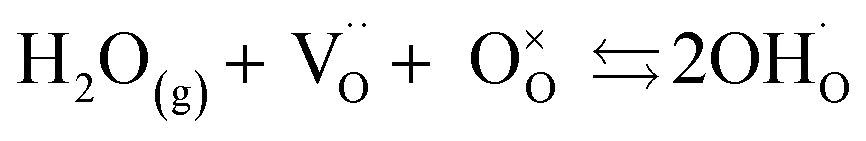
with 
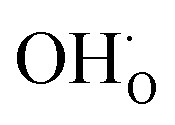
 corresponding to a OH^–^ ion sitting on a O lattice site with a positive charge.

However, in oxidizing conditions and in some systems, electronic holes can instead compensate for the vacancies *via* the following equation leading to p-type hole (h˙) conduction:3
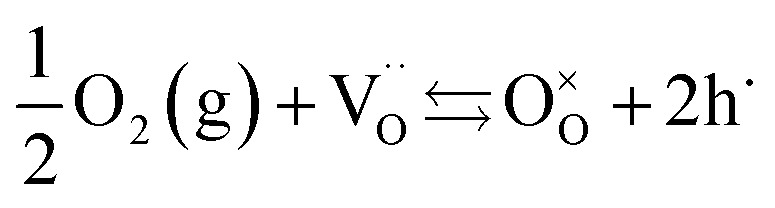



Under low oxygen partial pressures the following mechanism can occur yielding n-type electronic conduction:4
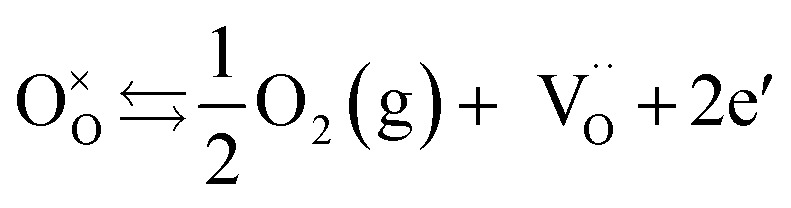



Significant proton conduction has been reported in substituted stannate phases such as BaIn_0.5_Sn_0.5_O_2.75_,^8^ Ba_2_YSnO_5.5_,^9^ BaSn_1–*x*_M_*x*_O_3–*δ*_ with M = Sc, Y, In and Gd, *x* = 0.125 (ref. 10) and *x* = 0.25 (ref. 11) and BaSn_1–*x*_Y_*x*_O_3–*δ*_ (0 ≤ *x* ≤ 0.5).^12^ More recently, Li and Nino reported on proton conductivity of BaSn_0.9_M_0.1_O_3–*δ*_ (M = In, Lu, Er and Y) in oxidising and reducing conditions,^13^ whilst Bévillon *et al.* ^14^ used a density functional theory approach to probe the energy landscape of the proton in substituted BaSn_1–*x*_M_*x*_O_3–*x*/2_.

In this study, BaSn_1–*x*_Sc_*x*_O_3–*δ*_ was selected as the system of interest as the recent studies highlighted above have established acceptor doped BaSnO_3_ as a promising alternative candidate to the more widely studied BaZrO_3_ and BaCeO_3_ systems. Scandium was chosen here as the dopant as it has an ionic radius that is only slightly larger than that of tin (0.745 Å and 0.69 Å for Sc^3+^ and Sn^4+^ respectively in 6-fold coordination).^15^ We report the preparation and characterisation of the BaSn_1–*x*_Sc_*x*_O_3–*δ*_ series with 0 ≤ *x* ≤ 0.4 *via* PXRD and solid-state NMR techniques, and a more detailed study of the highest accepter doped sample, BaSn_0.6_Sc_0.4_O_3–*δ*_. While the location of the oxygen vacancies was determined by ^119^Sn, ^45^Sc, and ^17^O multinuclear solid-state NMR spectroscopy by investigating the presence of Sn and Sc cations with various coordination numbers, the position of the deuteron ions in D_2_O treated BaSn_0.6_Sc_0.4_O_3–*δ*_ was found by neutron powder diffraction (NPD). Finally, the electrical conductivity was studied using electrochemical impedance spectroscopy (EIS) recorded under different atmospheres to reveal the temperature dependence of the dominating charge carriers.

## 2. Experimental

### 2.1. Synthesis

BaSn_1–*x*_Sc_*x*_O_3–*δ*_ with *x* = 0, 0.1, 0.2, 0.3 and 0.4 were synthesized by a solid state reaction using stoichiometric amounts of BaCO_3_ (Merck 99%), SnO_2_ (Sigma-Aldrich 99.9%), and Sc_2_O_3_ (Sigma-Aldrich 99.9%). The reactants were weighed and finely mixed to a paste using a mortar and pestle and ethanol before heating at 1000 °C for 8 h. The powders were then ball milled to a fine powder for 8 h in a Teflon milling house with ethanol using a planetary ball mill and zirconium milling balls. The powders were then dried and pressed into pellets, and subsequently reacted at 1200 °C for 72 h before being ball milled, pelletized and heated again at 1455 °C for 24 h. The sintered pellets were thereafter milled into a fine powder to give the as-prepared samples. All the heating steps were performed under an oxygen gas flow.

Hydration of BaSn_1–*x*_Sc_*x*_O_3–*δ*_ was performed by heating the powders with a stoichiometric amount of D_2_O, calculated to correspond to the complete filling of oxygen vacancies, in a hydrothermal bomb at 225 °C for 12 h. Drying of samples for NMR and NPD measurements was performed by treating at 900 °C for 8 h under vacuum. ^17^O NMR data were collected on samples that have been enriched in ^17^O by heating the freshly dried samples (1 h at 950 °C under vacuum) under 50% ^17^O enriched O_2_ gas (Isotec, 99%) for 2 days at 950 °C.

Conductivity measurements on a sample of BaSn_0.6_Sc_0.4_O_3–*δ*_ were performed on a 16 mm diameter, 72% dense pellet (made by uni-axially pressing of powders at 8 tons) which was sintered at 1455 °C for 24 h. The pellet was then coated on both faces with platinum paste, heated for 2 h at 1000 °C to remove the organic component of the paste, and finally, treated for 7 days in a furnace at 300 °C with a vapour saturated N_2_ gas flow (*p*(H_2_O) ≈ 0.40 atm) to give a pre-hydrated sample.

### 2.2. X-ray powder diffraction (PXRD)

PXRD data for the as-prepared samples were collected on a Bruker AXS D8 ADVANCE VARIO X-ray powder diffractometer (CuK_α1_ = 1.54058 Å) equipped with a LynxEye detector and a germanium (111) primary monochromator. The step size used was 0.050° with a collection time of 0.7 s per step in the 27° to 72° 2-theta range.

### 2.3. Neutron powder diffraction (NPD)

NPD data were collected at room temperature on dried BaSnO_3_, dried BaSn_0.6_Sc_0.4_O_3–*δ*_ and D_2_O treated BaSn_0.6_Sc_0.4_O_3–*δ*_ samples using the Polaris^16^ instrument at the ISIS neutron facility, and subsequently analysed using the GSAS^17,18^ software package. Data from two detector banks were used for the structure refinements, *i.e.* the backscattering detector bank covering scattering angles of 130° < 2*θ* < 160°, and a *d*-spacing range of 0.2 < *d* (Å) < 3.2, with a resolution of Δ*d*/*d* ∼ 5 × 10^–3^, and the 90° detector bank (85° < 2*θ* < 95°; 0.3 < *d* (Å) < 4.1; Δ*d*/*d* ∼ 7 × 10^–3^). Data were collected for approximately 9 h for the D_2_O treated BaSn_0.6_Sc_0.4_O_3–*δ*_ sample and 1 h for the dried samples.

Rietveld refinements^18–20^ included the following parameters: a scale factor, the cubic lattice parameter *a*, background parameters describing a reciprocal interpolar function, isotropic thermal vibration parameters for the cation sites, *u*_Ba_, *u*_Sn/Sc_, and anisotropic parameters for the oxygen site, *u*_11_, *u*_22_ = *u*_33_ and 4 profile parameters describing Gaussian and Lorentzian contributions to the Bragg peak profiles in the cubic space group *Pm*3*m*. Ba was set at 1b (½, ½, ½), Sn/Sc at 1a (0, 0, 0) and O at 3d (½, 0, 0). Ahmed *et al.* ^21^ reported the likelihood of the deuteron being located at the 24k (0.55, 0.20, 0) crystallographic site for BaZr_0.5_In_0.5_O_2.5_(OD)_0.5_ and this was used as a starting point in the analysis of the data from the deuterium containing sample.

### 2.4. Solid-state NMR

^119^Sn NMR spectra were acquired at 11.7 T on a wide bore Oxford 500 MHz Varian Infinity Plus spectrometer using a 3.2 mm HX Chemagnetics probehead tuned to 186.26 MHz. The BaSnO_3_ (dried) and BaSn_1–*x*_Sc_*x*_O_3–*δ*_ samples (in vacuum dried and D_2_O treated forms) were packed under nitrogen gas atmosphere in 3.2 mm zirconia rotors, which were then spun at a spinning frequency *ν*_r_ = 20 kHz. ^119^Sn single pulse experiments were carried out using a π/2 pulse width of 2 μs (*i.e.* at an rf field amplitude of *ν*Sn1 = 125 kHz) and a recycle delay of 70 s allowing full relaxation of the ^119^Sn spins. Chemical shifts were externally referenced to SnO_2_ at –604.3 ppm.

High field ^45^Sc NMR experiments were performed at 19.6 T on a ultra-narrow bore Bruker DRX 830 MHz spectrometer at the National High Magnetic Field Laboratory, Tallahassee, Florida, USA using a home-built 1.8 mm single channel probe^22^ tuned to 202.44 MHz. All samples were packed inside 1.8 mm rotors, spun at a spinning frequency *ν*_r_ of 33.333 kHz, and short recycle delays of 0.2 s allowing full relaxation of the ^45^Sc spins were used for the 1D spectra. *t*_1_ rotor synchronized two-dimensional (2D) triple-quantum MAS (TQMAS) experiments^23–25^ were performed using a shifted-echo pulse sequence and the Soft-Pulse-Added-Mixing (SPAM) enhancement pulse.^26^ Hard and soft pulses were performed at radio-frequency (rf) field amplitudes of *ν*Sc1 = 150 kHz and approximately *ν*Sc1 = 20 kHz, respectively. Chemical shifts were externally referenced to a 1 M solution of Sc(NO_3_)_3_ in water at 0.0 ppm.

^17^O NMR experiments were carried out on a 17.6 T wide bore Bruker Avance 750 MHz spectrometer equipped with a 4 mm HXY (in double resonance mode) probehead and operating at 101.72 MHz. All samples were packed inside 4 mm rotors and spun at a spinning frequency *ν*_r_ of 15 kHz. ^17^O one-dimensional spectra were recorded using a one pulse sequence with selective pulse widths of π/6 = 0.6 μs and at an rf field amplitude of *ν*O1 = 50 kHz. *t*_1_ rotor synchronized two-dimensional (2D) TQMAS experiments were performed using the *z*-filtered pulse sequence.^27^ Hard and soft pulses were performed at rf field amplitudes of *ν*O1 = 50 kHz and approximately *ν*O1 = 10 kHz, respectively. The recycle delays were set to 5 s for all experiments. Chemical shifts were externally referenced to water at 0.0 ppm.

All data were processed with MatLab and MatNMR.^28^

### 2.5. Impedance spectroscopy

A ProboStat™ (NorECs AS, Norway) cell coupled to a Solartron 1260 frequency response analyser in standalone mode was used to collect electrochemical impedance data. Data collection was between 1 Hz and 1 MHz at 1 V rms amplitude between 150 and 1000 °C in steps of 50 °C with an equilibration time of 30 minutes before data collection. Data was collected for BaSn_0.6_Sc_0.4_O_3–*δ*_ in the following sequence: pre-hydrated sample heating and cooling in dry Ar gas, wet (humidified) Ar gas cooling, wet O_2_ gas on cooling, and finally dry O_2_ gas on cooling. Two silica tubes, one inside the other, were used to cover the cell, and two P_2_O_5_ gas traps before the cell were used to ensure dry gas conditions within the cell. A dense mullite–alumina tube was used in conjunction with a water bubbler at ambient temperature to provide wet gas (*p*(H_2_O) ≈ 0.025 atm) within the cell.

## 3. Results

### 3.1. X-ray diffraction

**Fig. 1:**
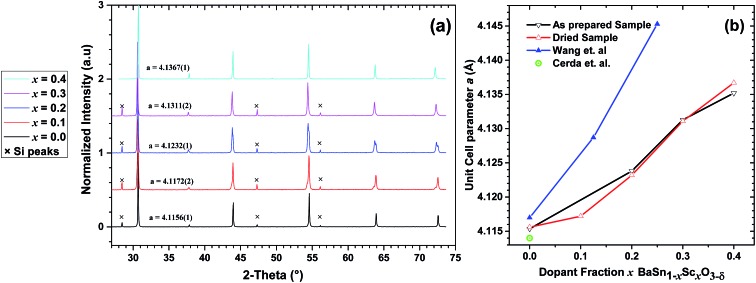
(a) PXRD patterns collected on dried BaSn_1–*x*_Sc_*x*_O_3–*δ*_ samples with the indicated cell parameters in Ångstroms. (b) Comparison of the cell parameters for the dried and as prepared BaSn_1–*x*_Sc_*x*_O_3–*δ*_ samples with values reported by Wang *et al.*,^10,11^ and Cerda *et al.* ^30^

Fig. 1 shows the PXRD pattern for all dried BaSn_1–*x*_Sc_*x*_O_3–*δ*_ samples (*x* = 0.0, 0.1, 0.2, 0.3 and 0.4). These data reveal that all samples are highly crystalline and the patterns indicate that the phases adopt a cubic perovskite structure (space group *Pm*3*m*) across the range of compositions. The cell parameters, obtained from profile fitting using Jana2006,^29^ increased with increase of dopant fraction in agreement with the Sc^3+^ ionic radius (0.745 Å) being larger than Sn^4+^ (0.69 Å).^15^ Close inspection of the data revealed evidence of peak shoulders at 2*θ* ≈ 44°, 54° for the *x* = 0.1, 0.2 and 0.3 samples. This behaviour was rationalised in terms of phase segregation into BaSnO_3_ and a BaSn_1–*x*_Sc_*x*_O_3–*δ*_ phase comparatively rich in scandium in order to preserve the overall stoichiometry of the initial sample reactants. This behaviour was not apparent for the *x* = 0.4 sample, and its cell parameter of 4.1367(1) Å showed a significant enlargement compared to the value of 4.1156(1) Å determined for un-doped BaSnO_3_. BaSn_0.6_Sc_0.4_O_3–*δ*_, which showed the highest incorporation of scandium based on the PXRD results, was therefore selected for further study *via* neutron diffraction and impedance measurements.

### 3.2. Neutron diffraction

**Fig. 2:**
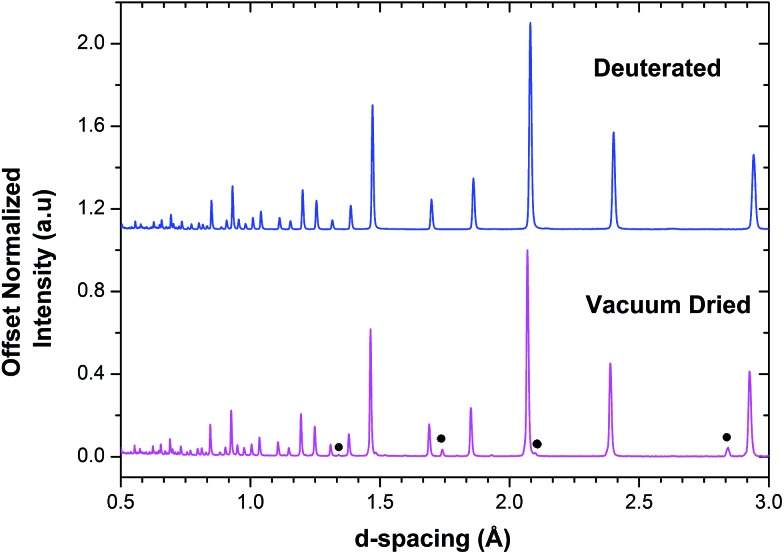
NPD patterns of vacuum dried and deuterated BaSn_0.6_Sc_0.4_O_3–*δ*_. Black filled circles indicate reflections arising from a small amount of Sc_2_O_3_.

The NPD data for BaSn_0.6_Sc_0.4_O_3–*δ*_ presented in Fig. 2 revealed that a minor Sc_2_O_3_ impurity phase was present in the vacuum dried sample. The large neutron scattering lengths of scandium (12.29 fm) and oxygen (5.803 fm) compared to their relatively weaker X-ray scattering powers could explain why this minor phase was detected in the neutron pattern (Fig. 2) but was not visible in the PXRD patterns (Fig. 1). The Sc_2_O_3_ peaks are not visible in the NPD of deuterated BaSn_0.6_Sc_0.4_O_3–*δ*_ where only a single, deuteron containing, perovskite phase is present.

#### 3.2.1. Vacuum dried BaSn_0.6_Sc_0.4_O_3–*δ*_

As starting models three phases namely, BaSn_0.6_Sc_0.4_O_2.8_ and BaSnO_3_, both modelled using the cubic *Pm*3*m* crystal system, and Sc_2_O_3_ were included into the Rietveld analysis of the dried sample, phase 1, 2 and 3 respectively. The weight fractions of these three phases obtained from the refinement were 95.55(1) wt%, 3.34(8) wt% and 1.11(1) wt%, respectively (Fig. S1†). The Sn : Sc site occupancy in the dominant perovskite phase 1 was reciprocally linked and refined to give a small increase in the Sn : Sc ratio, with 0.666(2) and 0.334(2) site occupancies for Sn and Sc, respectively. The overall sample stoichiometry was consistent with the initial 0.6 Sn and 0.4 Sc molar fractions. Modelling the oxygen atoms with an anisotropic displacement parameter (ADP) significantly reduced the values of the *χ* ^2^ goodness of fit parameter from 15.66 to 10.86. Simultaneous refinement of the oxygen ADP and occupancy was not deemed reliable due to the high degree of correlation between these two variables. Hence the occupancy of the oxygen site in phase 1 was set to 0.944 as would be expected for a dried sample with a BaSn_0.666_Sc_0.334_O_3–*δ*_ composition with a final *χ* ^2^ value of 6.372. The final agreement to the data is shown in the supplementary data (Fig. S1†). Note that for simplicity we continue to refer to this sample as BaSn_0.6_Sc_0.4_O_3–*δ*_, despite the slightly lower Sc content of the perovskite phase.

#### 3.2.2. Hydrothermally D_2_O treated BaSn_0.6_Sc_0.4_O_3–*δ*_

**Fig. 3:**
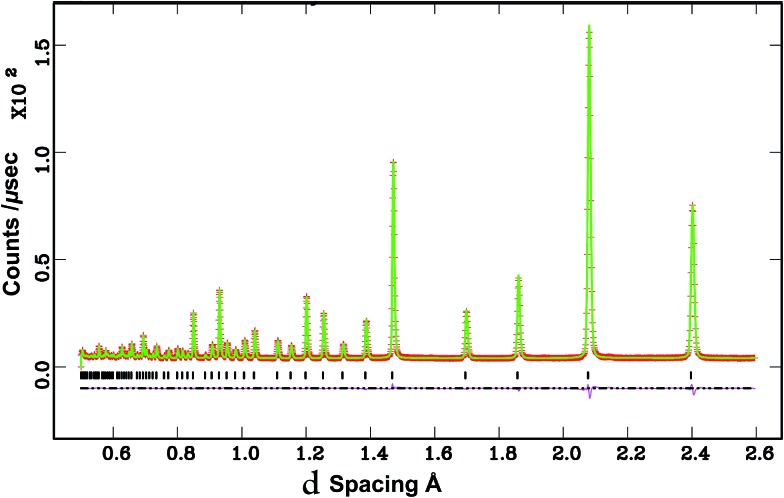
Rietveld fit to the data for BaSn_0.6_Sc_0.4_O_2.6_(OD)_0.4_ with the deuteron modelled at the 24k site. Both the observed (red crosses) and calculated (continuous green line) profiles are plotted and the position of the reflections is marked with vertical black bars. The difference curve lies at the bottom in purple.

Given the absence of additional impurity reflections, the Sn^4+^ and Sc^3+^ occupancies used in the Rietveld fit to the NPD data were fixed to the nominal values of 0.6 and 0.4, respectively, for D_2_O treated BaSn_0.6_Sc_0.4_O_3–*δ*_. The occupancy of the oxygen and barium sites was permitted to vary and both favoured a value slightly above unity and were therefore set to one. This is consistent with complete filling of oxygen vacancies by OD groups as per eqn (2) above during the hydrothermal treatment with D_2_O. The fit improved significantly by allowing the oxygen ADP factor to vary anisotropically. The deuteron position was investigated by Rietveld analysis and the use of Fourier difference maps as described previously.^21^ Missing positive scattering was observed near fractional coordinates *x* = 0.55, *y* = 0.2 and *z* = 0.0, *i.e.* the crystallographic 24k site. The deuteron site occupancy was calculated from the number of filled oxygen vacancies with respect to the refined oxygen occupancy, *e.g.* BaSn_0.6_Sc_0.4_O_2.6_(OD)_0.4_, which corresponds to a 24k site occupancy of ∼0.017. The isotropic ADP parameter was then set free to refine together with the atomic coordinates *x* and *y* of the deuteron at the 24k site. This resulted in a significant reduction in the standard uncertainties of the refined parameters and a small reduction in the goodness of fit parameters. The deuteron positional coordinates (*x*, *y*, 0) refined to (0.579(3), 0.217(3), 0). Results of the Rietveld analysis of the NPD data are listed in Table 1, and the final Rietveld fit achieved is shown in Fig. 3.

**Table 1:** Refined parameters from neutron powder diffraction of dried BaSnO_3_ and both dried and deuterated BaSn_0.6_Sc_0.4_O_3–*δ*_

Refinement parameters	Dry BaSnO_3_	BaSn_0.6_Sc_0.4_O_3–*δ*_	
		Dry	Deuterated
*a* (Å)	4.11588(2)	4.13549(1)	4.15716(2)

***U***_**iso**_ **(Å** ^**2**^ **) × 100**			
Ba	0.416(4)	0.614(5)	1.200(5)
Sn/Sc	0.231(3)	0.712(6)	1.057(4)
O *U*_11_	0.294(8)	0.334(11)	0.498(8)
O *U*_22_–*U*_33_	0.923(6)	1.201(7)	1.261(5)
D (*x*, *y*, 0)			0.579(3), 0.217(3), 0
D (*x*, *y*, 0) *U*_iso_			11.3(4)

**Occupancy**			
Ba (0.5, 0.5, 0)	1.0^*a*^	1.0^*a*^	1.0^*a*^
Sn (0, 0, 0)	1.0^*a*^	0.666(2)	0.6
Sc (0, 0, 0)	—	0.334(2)	0.4
O (0.5, 0, 0)	1.0	0.944^*b*^	1.0^*a*^
D (*x*, *y*, 0)	—	—	0.0167^*c*^

**Bond distances (Å)**			
12 × Ba–O	2.91037(1)	2.92423(1)	2.93956(1)
6 × Sn/Sc–O	2.05794(1)	2.06774(1)	2.07858(1)
1 × O–D	—		0.959(12)
1 × O–D^(1–*y*, *x*, *z*)^			2.109(7)
1 × O–D^(–*y*, *x*, *z*)^			2.680(6)
2 × O–D^(1+*z*, *y*, –*x*)^			2.862(9)
*χ* ^2^	48.16	6.37	12.84
*R*_wp_ (%)	0.0314	0.0283	0.0124
*R*_p_ (%)	0.0360	0.0441	0.0169

**Phases (wt%)**			
Main	100	95.55(1)	100
Sc_2_O_3_	—	1.11(1)	—
BaSnO_3_	—	3.34(8)^*d*^	—
Variables	—	26	47

^*a*^Occupancies refined to slightly larger than 1, and therefore fixed to 1.

^*b*^Due to correlation between the oxygen site ADP and occupancy this value was not refined in final stages, instead it was fixed to the value determined by the amount of refined scandium *x* = 0.334 (oxygen fraction = (3 – (0.334/2))/3).

^*c*^Occupancy of the deuterium site was fixed to reflect a deuterium content consistent with complete filling of the oxygen vacancies.

^*d*^The refined unit cell parameter was 4.11457(17) Å.

### 3.3. Solid-state NMR

#### 3.3.1. ^119^Sn NMR

The ^119^Sn magic angle spinning (MAS) NMR spectrum of dry BaSnO_3_ (Fig. S2a in the ESI†) shows a sharp resonance at –679 ppm corresponding to tin in a six-fold symmetrical environment.^31^ This environment corresponds to Sn surrounded by 6 tin atoms in its first cation coordination shell, giving rise to a Sn(OSn)_6_ local environment,^31^ the only chemical environment in the undoped BaSnO_3_ material.

**Fig. 4:**
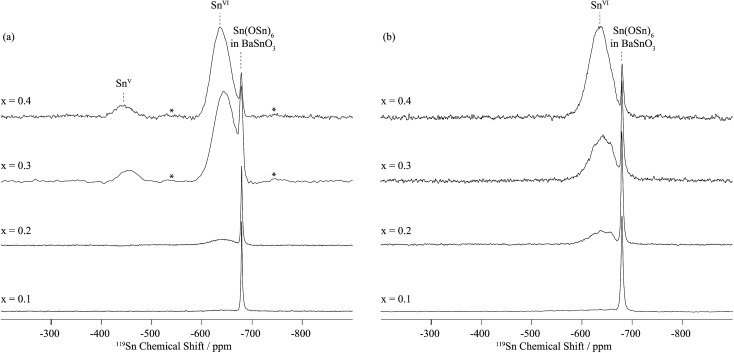
^119^Sn MAS NMR single pulse spectra of (a) dry BaSn_1–*x*_Sc_*x*_O_3–*δ*_ and (b) deuterated BaSn_1–*x*_Sc_*x*_O_3–*δ*_ as a function of Sc doping level *x* were obtained at 11.7 T and under a MAS frequency of 20 kHz. Sn^VI^ and Sn^V^ denote six and five coordinated tin environments. Asterisks (*) indicate spinning side bands.

In dry Sc-substituted BaSnO_3_, a new set of ^119^Sn resonances with intensity proportional to Sc concentration appears at around –640 ppm (Fig. 4a). This feature is assigned to tin in six fold environments surrounded by at least one scandium cation based on previous NMR studies of the related Y-doped BaSnO_3_ materials.^32^ In this system, the six-coordinated Sn cations with various numbers of Y ions in their first cationic coordination shells, *i.e.* Sn(OSn)_5_(OY), Sn(OSn)_4_(OY)_2_, Sn(OSn)_3_(OY)_3_, *etc.* could be individually observed, the ^119^Sn resonance shifting by +27 to +34 ppm per added Y ion. Here the ^119^Sn spectra of Sc-doped BaSnO_3_ lacks such sharp, resolved features, most likely due to a smaller frequency shift per Sc ion added to the vicinity of the Sn nuclei. Indeed the ionic radius of Sc^3+^ in 6-fold coordination (0.745 Å) is much closer to the one of Sn^4+^ (0.69 Å) in comparison to Y^3+^ (0.90 Å) leading to smaller local distortions in the case of Sc substitution and therefore smaller frequency shifts. A weak but sharp BaSnO_3_ resonance is seen in all four samples (*x* = 0.1, 0.2, 0.3 and 0.4) at –679 ppm most likely due to a separate BaSnO_3_ impurity phase; the weakest BaSnO_3_ resonance was seen for *x* = 0.4, consistent with the low phase fraction obtained in the NPD refinement of this phase (Fig. 2 and S1†).

A second broad resonance, centered at –450 ppm (Fig. 4a), is assigned to five-coordinated Sn environments in line with a shift to higher frequency going from six to five-fold Sn coordination,^31,32^ a trend generally observed for a number of nuclei.^33^

On hydrothermal D_2_O treatment of dry BaSn_1–*x*_Sc_*x*_O_3–*δ*_ (Fig. 4b), the ^119^Sn NMR resonance at –450 ppm, associated with the five-coordinated Sn environments, totally disappears, which is consistent with its assignment, and shows complete reaction of the oxygen vacancies 
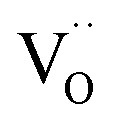
 with D_2_O during hydration to form six-coordinated Sn environments (experimentally observed at –636 ppm). The sharp –679 ppm resonance is also seen, providing evidence for BaSnO_3_ environments although no sign of an impurity phase was detected in the NPD data, presumably due to its low concentration.

#### 3.3.2. ^45^Sc NMR

**Fig. 5:**
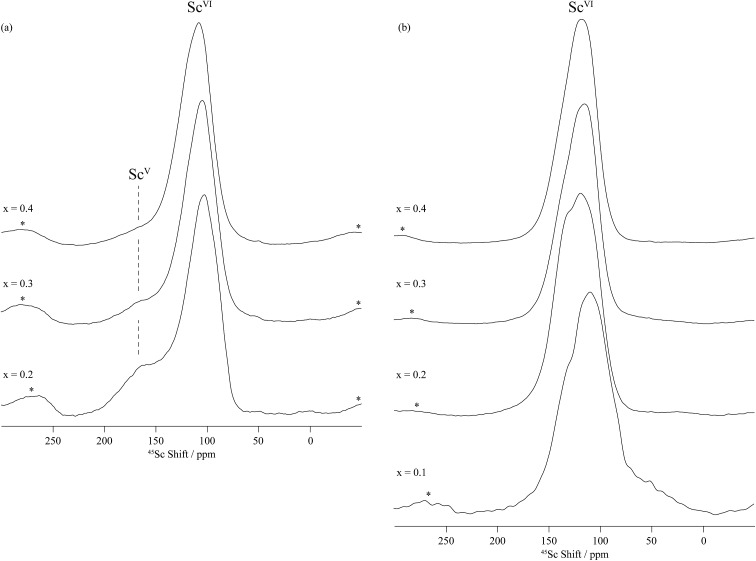
Central transition ^45^Sc MAS NMR spectra of (a) dry BaSn_1–*x*_Sc_*x*_O_3–*δ*_ and (b) deuterated BaSn_1–*x*_Sc_*x*_O_3–*δ*_ as a function of Sc doping level *x*. The spectra were obtained at 19.6 T and under MAS frequency of 33.33 kHz. The ^45^Sc MAS NMR spectra of dry BaSn_0.9_Sc_0.1_O_3–*δ*_ was not recorded. Sc^VI^ and Sc^V^ denote six and five coordinated scandium environments. Asterisks (*) indicate spinning side bands.

**Fig. 6:**
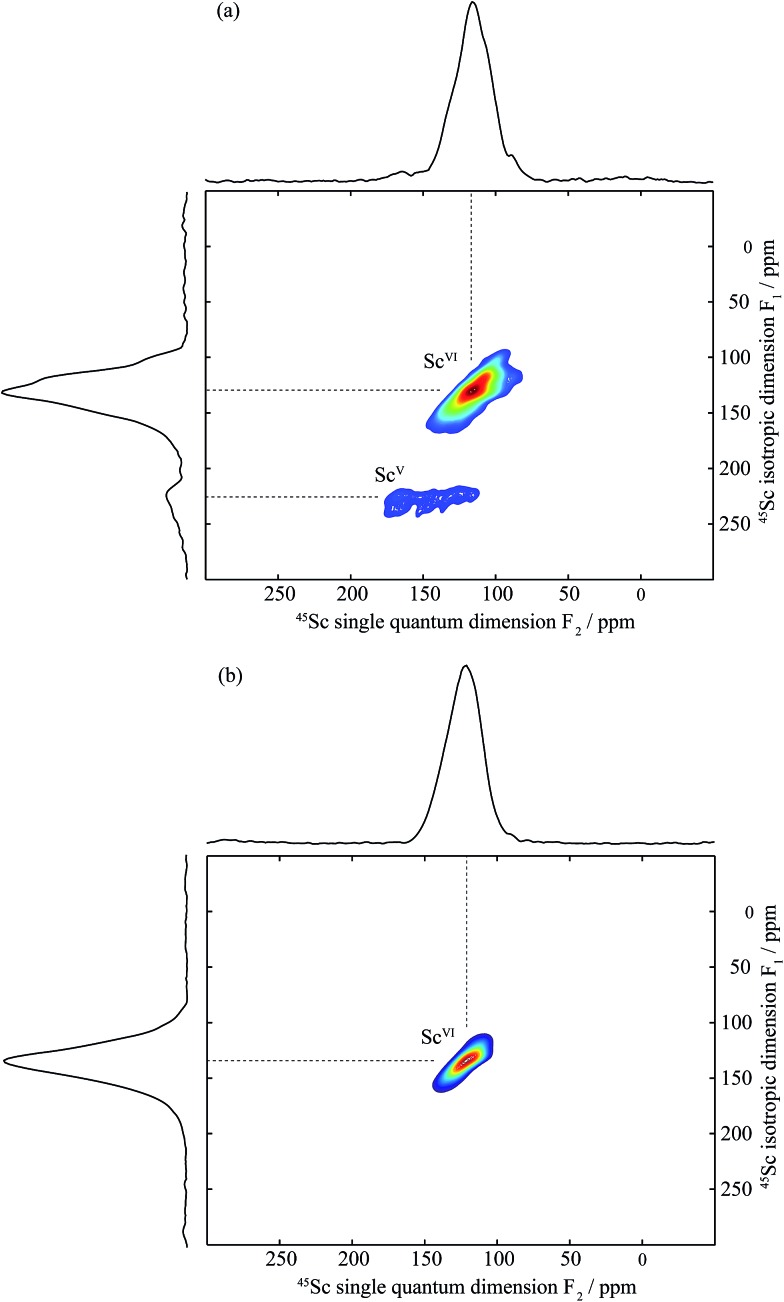
Two-dimensional sheared triple-quantum ^45^Sc MAS spectra of (a) dry BaSn_0.6_Sc_0.4_O_3–*δ*_ and (b) deuterated BaSn_0.6_Sc_0.4_O_3–*δ*_ obtained at 19.6 T with a MAS frequency of 33.33 kHz. 9600 transients were accumulated for each of the 24 (for (a)) and 16 (for (b)) *t*_1_ increments at a recycle delay of 0.4 s. Top: anisotropic skyline projection (the ^45^Sc MAS NMR single pulse spectra are given in Fig. 5 and S3†). Left: isotropic skyline projection of the TQMAS spectra. Sc^VI^ and Sc^V^ denote six and five coordinated scandium environments, respectively.

Fig. 5 and S3 (in the ESI†) show the one-dimensional ^45^Sc spectra of dry and deuterated BaSn_1–*x*_Sc_*x*_O_3–*δ*_ as a function of Sc doping level obtained at a high magnetic field of 19.6 T under fast MAS. The spectra of the dry samples contain a main resonance centred at around 110 ppm in addition to a much broader resonance in the 200–100 ppm region, which disappears upon hydrothermal D_2_O treatment. Solid-state NMR spectra of quadrupolar nuclei such as ^45^Sc (spin = 7/2) are often broad even under MAS because of residual second-order quadrupolar interactions. This can be removed by performing a two-dimensional triple-quantum MAS experiment (TQMAS)^23–25^ whose vertical projection along the F_1_ dimension yields a one-dimensional isotropic spectrum free of second-order broadening. Such experiments have been recorded for the dry and deuterated BaSn_1–*x*_Sc_*x*_O_3–*δ*_ materials (Fig. 6, S4–S6†) and are discussed below.

Two sets of resonances are clearly observed in the F_1_ vertically projected spectra of the dry BaSn_0.6_Sc_0.4_O_3–*δ*_ sample at shifts of approximately 125 and 225 ppm (Fig. 6) demonstrating the presence of two different scandium environments. Extraction of the shifts of these resonances in the horizontal F_2_ dimension allow isotropic chemical shifts values of around 120 and 200 ppm to be extracted (see Table 2) and assigned to 6- and 5-coordinated scandium environments, respectively, based on previous study by Stebbins *et al.*,^34^ our previous work on the BaZr_1–*x*_Sc_*x*_O_3–*δ*_ series,^35^ and Takamura *et al.*'s recent hydration study of 10% mol Sc-substituted BaZrO_3_.^36^ The 5-coordinated Sc environment which has a very large linewidth (leading to a quadrupolar coupling of around 20 MHz) is ascribed to the presence of an oxygen vacancy in the 1^st^ coordination shell of a Sc atom.

**Table 2:** Experimental ^119^Sn, ^45^Sc and ^17^O NMR parameters dry BaSnO_3_, dry BaSn_0.6_Sc_0.4_O_3–*δ*_ and deuterated BaSn_0.6_Sc_0.4_O_3–*δ*_ ^*a*^

Site	Environment	*δ*_iso_/ppm	*C*_Q_/MHz	*η*_Q_
**Dry BaSnO**_**3**_				
Sn	Sn^VI^(OSn)_6_	–679	—^*b*^	
Sc	—^*b*^			
O^*c*^	Sn^VI^–O–Sn^VI^	152	6.1	0.0

**Dry BaSn**_**0.6**_ **Sc**_**0.4**_ **O**_**3–*δ***_				
Sn	Sn^VI^(OSn)_6_ in BaSnO_3_	–679(1)^31^	—^*b*^	
	Sn^VI^	–640(10)		
	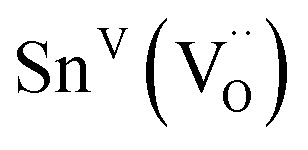	–450(10)		
Sc	Sc^VI^	120(5)	7(2)	0.7(3)
	Sc^V^	199(7)	20(2)	0.0(1)
O	Sn–O–Sn	203(5)	8(1)	—^*d*^
	Sn–O–Sc	248(10)	3(1)	—^*d*^
	Sn–O–Sc	261(8)	3(1)	—^*d*^
	Sc–O–Sc	420	5(1)	—^*d*^

**Hydrothermally D**_**2**_ **O treated BaSn**_**0.6**_ **Sc**_**0.4**_ **O**_**3–*δ***_				
Sn	Sn^VI^(OSn)_6_ in BaSnO_3_	–679(1)^31^	—^*b*^	
	Sn^VI^	–636(10)		
Sc	Sc^VI^	122(5)	7(2)	0.8(1)
O	—^*d*^			

^*a*^ ^45^Sc and ^17^O NMR parameters were determined from the peak positions in the TQMAS spectra at 19.6 and 17.6 T, respectively (see ESI).^25^

^*b*^Not applicable.

^*c*^Data from ref. 32.

^*d*^Not measured experimentally.

Upon hydrothermal D_2_O treatment, the oxygen vacancies are filled by protonic (deuterons) and OD defects resulting in the loss of the 5-coordinated Sc as revealed by the ^45^Sc NMR spectra given in Fig. 5b and S4–S6b.† These spectra are now dominated by resonances centered at around 125 ppm and corresponding to 6-coordinated Sc only (Table 2). In fact, more than one 6-coordinated Sc environments are often visible in the ^45^Sc MQMAS spectra of deuterated BaSn_1–*x*_Sc_*x*_O_3–*δ*_, and are assigned to ScO_6_ (as in the dry samples) and ScO_5_(OD) environments (*i.e.* 6-coordinated scandium in the vicinity of a protonic defect).

Note that the Sc_2_O_3_ impurity seen by NPD was not observed by ^45^Sc NMR of dry BaSn_0.6_Sc_0.4_O_3–*δ*_ (Fig. 6a). This is attributed to the very small amount of Sc_2_O_3_ (1.11%, Table 1), which is probably below the NMR detection limit, and to the fact that the two 6-coordinated Sc sites in Sc_2_O_3_ have isotropic chemical shifts of 108 and 128 ppm,^34^ very close to the value for 6-coordinated Sc in this sample. The lack of resolution might therefore also prevent its observation.

#### 3.3.3. ^17^O NMR

**Fig. 7:**
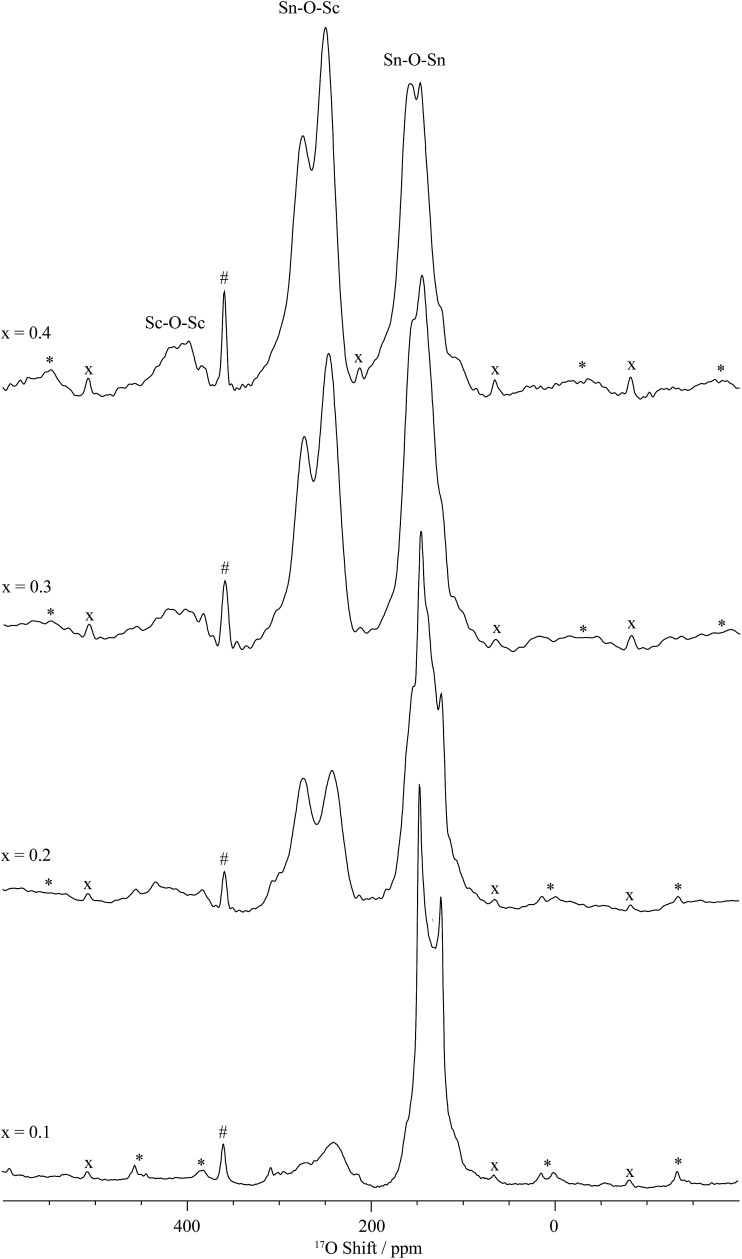
Central transition ^17^O MAS NMR single pulse spectra of ^17^O enriched BaSn_1–*x*_Sc_*x*_O_3–*δ*_ as a function of the Sc doping level *x*. The spectra were obtained at 17.6 T and under MAS frequency of 15 kHz. The asterisks (*), dash (#) and crosses (×) denote spinning side bands, the ^17^O signal of the ZrO_2_ rotor and its spinning side bands, respectively.

^17^O NMR is usually very challenging due to the very low natural abundance of ^17^O (approximately 0.037%), often requiring isotopic enrichment. This is routinely performed in these materials *via* a gas–solid exchange reaction with ^17^O enriched O_2_ gas^37,38^ at elevated temperatures (see the Experimental section for further details). All the one-dimensional ^17^O MAS NMR spectra of ^17^O enriched BaSn_1–*x*_Sc_*x*_O_3–*δ*_ (Fig. 7 and S7†) show three sets of resonances, at around 150 ppm, in the 200–300 ppm region, and centred at around 420 ppm (Table 2). The resonance around 150 ppm region consists of a single oxygen environment (see the ^17^O MQMAS spectra, Fig. S8†) and is assigned to a bridging oxygen bound to two tin cations, *i.e.*, Sn–O–Sn, based on our previous work on BaSn_1–*x*_Y_*x*_O_3–*δ*_.^32^ As the Sc content is increased, this resonance remains strongly present (see Fig. 7 for BaSn_0.6_Sc_0.4_O_3–*δ*_) indicating the lack of significant Sn/Sc ordering in this material. Two peaks are clearly observed in the 200–300 ppm region, their intensities increasing with Sc concentration (relative to those for Sn–O–Sn); they are both tentatively assigned to Sc–O–Sn environments, the presence of two resonances possibly arising from Sc and Sn being five or six coordinated. A third broad resonance centred at around 420 ppm is also observed and is assigned to Sc–O–Sc oxygen environments based on previous work on the related Sc doped BaZrO_3_ cubic perovskite.^35^

### 3.4. Impedance spectroscopy

**Fig. 8:**
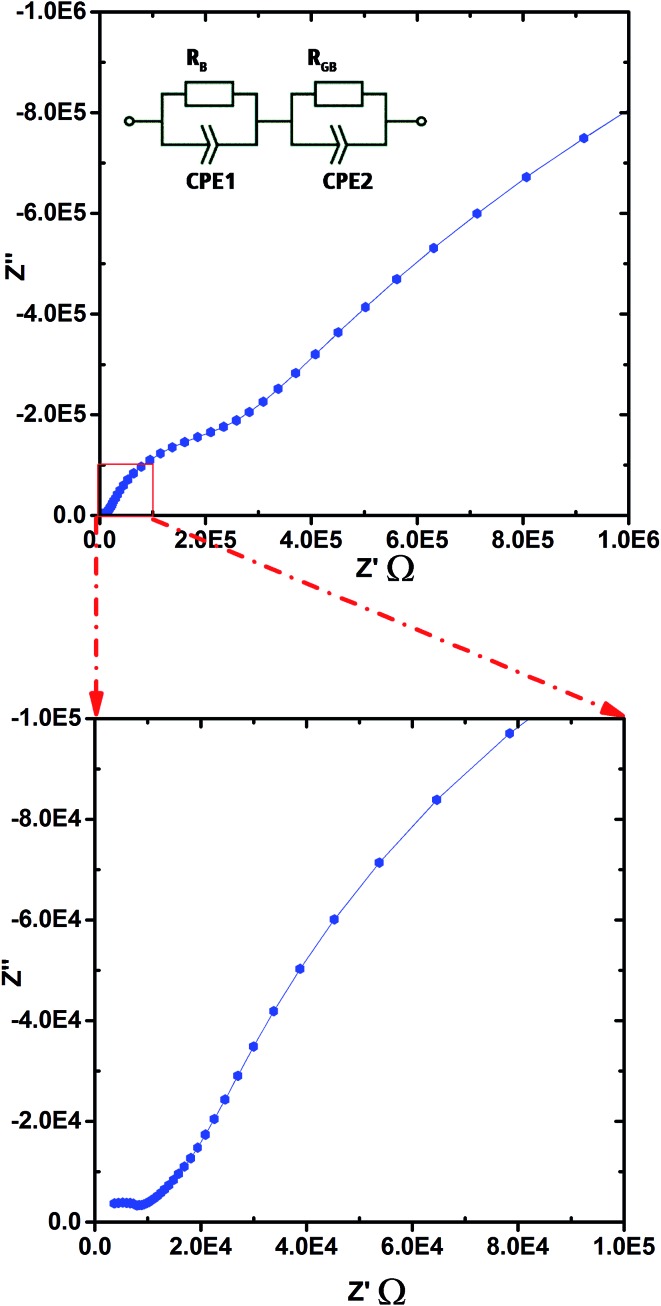
Typical Nyquist plot of the impedance showing the full 1 Hz to 1 MHz range for pre-hydrated BaSn_0.6_Sc_0.4_O_3–*δ*_ on heating in a dry Ar atmosphere at 100 °C, the blue lines representing visual guides. A magnified view at low *Z*′ is shown in the insert.

Fig. 8 shows the complex plane plot of the pre-hydrated BaSn_0.6_Sc_0.4_O_3–*δ*_ sample on heating at 100 °C in dry Ar. Two time constants are observed, including one in the high frequency region near the origin; the data is modelled using two (RQ) elements, representing a resistor and constant phase element in parallel, connected in series. The derived capacitances were 1.17 × 10^–11^ F cm^–2^ and 8.14 × 10^–9^ F cm^–2^ consistent with bulk and grain boundary processes, respectively. The feature at the lowest frequencies is attributed to electrode processes. For the initial heating and cooling data, it was possible to separate bulk and grain boundary conductivity in this manner at temperatures below approximately 400 °C. At higher temperatures, and for the other atmospheres and thermal protocols, the data were analysed using a similar approach but here the distinction between bulk and grain boundary was not as clear and only the total conductivity (bulk + grain boundary) could be extracted.

**Fig. 9:**
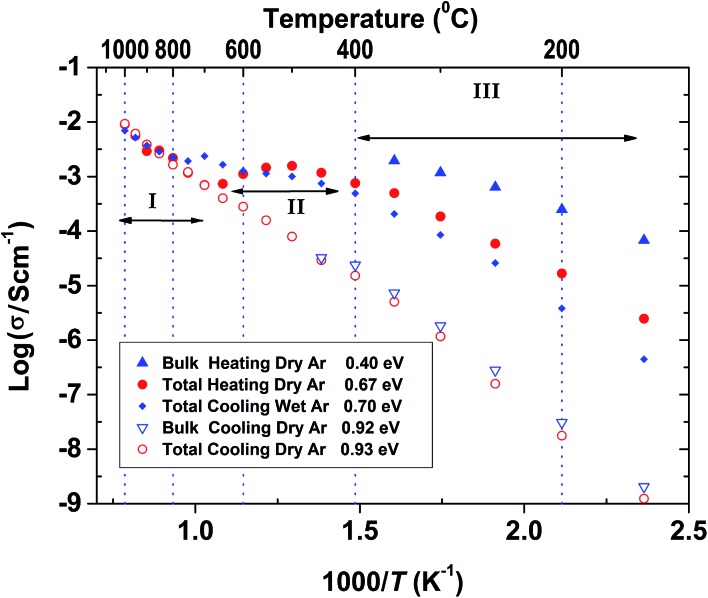
Arrhenius plot of the conductivity of an initially hydrated BaSn_0.6_Sc_0.4_O_3–*δ*_ sample under dry or wet Ar gas atmospheres. Three conductivity regimes (I, II and III) are observed, as highlighted, when the sample contains protons. The activation energies in the temperature regime III (below 400 °C) are indicated in the inserts.

**Fig. 10:**
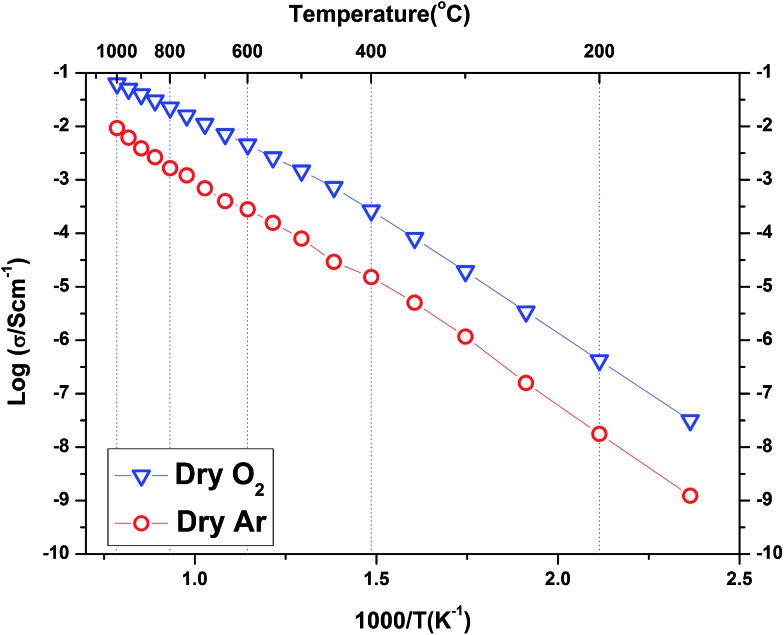
A comparison of the total conductivity of BaSn_0.6_Sc_0.4_O_3–*δ*_ under dry Ar gas and dry O_2_ gas. The activation energies below 400 °C were 0.95 eV and 0.90 eV respectively.

**Fig. 11:**
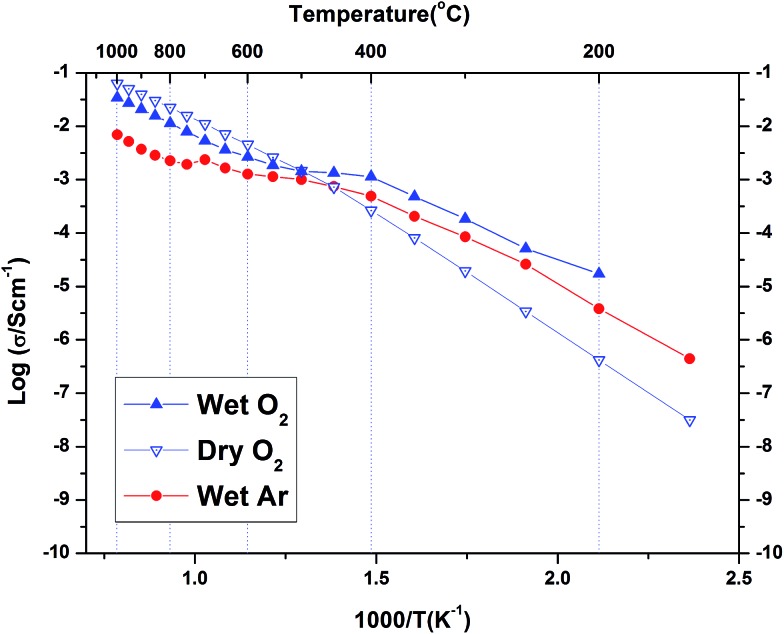
Total conductivity under dry O_2_, wet O_2_ and wet Ar gas conditions. The activation energy for the conduction processes were 0.61 eV and 0.64 eV in dry and wet O_2_ respectively in the region above 600 °C while below 400 °C they were 0.90 eV and 0.63 eV respectively.

The conductivity data collected for BaSn_0.6_Sc_0.4_O_3–*δ*_ under Ar consisted of three regions (see Fig. 9). Region I between 800–1000 °C has O^2–^ anions or possibly electron holes as the dominant charge carriers, while region II, between 400 and 800 °C is characteristic of the growing influence of protons and displays a characteristic plateau^39^ that reflects the simultaneously varying proton concentration and proton mobility. Region III, at *T* ≤ 400 °C, is dominated by proton charge carriers. Comparison of the conductivity under dry Ar *vs.* dry O_2_ conditions reveals that the sample possesses significantly higher conductivity, approximately one order of magnitude greater, under oxidizing conditions throughout the entire temperature interval as evident in Fig. 10. Conductivity under wet oxygen and above 450 °C was found, unexpectedly, to be lower than that in dry oxygen (Fig. 11).

## 4. Discussion

### 4.1. Phase formation

The cell parameters extracted from the PXRD results of the as-synthesized BaSn_1–*x*_Sc_*x*_O_3–*δ*_ (*x* = 0.0, 0.1, 0.2, 0.3, 0.4) samples were found to increase with the level of Sc^3+^ dopant content. This expansion is expected as it reflects the greater ionic radius of Sc^3+^ (0.745 Å, 6-fold coordination) in comparison to Sn^4+^ (0.69 Å).^15^ Additionally, samples with dopant concentrations in the range *x* = 0.1–0.3, contained peak shoulders in their XRD patterns, which were attributed to a level of phase segregation. These results were in agreement with the ^119^Sn NMR data which revealed the presence of sharp Sn(OSn)_6_ environments similar to that observed in BaSnO_3_ (Fig. S2†).

The cell parameters obtained in this paper for BaSn_1–*x*_Sc_*x*_O_3–*δ*_ are visibly lower than those reported by Wang and co-workers,^10,11^ (Fig. 1b) but for BaSnO_3_ our reported value is still higher than the value of 4.1140 Å reported by both Roth *et al.* ^40^ and Cerda *et al.* ^30^ Anomalous behaviour has been reported for Sc^3+^ doped BaZrO_3_ (ref. 41) system with samples sintered at a lower temperature having a larger unit cell parameter when compared to samples sintered at higher temperatures. Hiraiwa *et al.* ^41^ demonstrate this behaviour is unique to the Sc^3+^ dopant and is contrary to the behaviour of other dopants for BaZrO_3_. However no hypothesis exists yet to explain this behaviour.

Although BaSn_0.6_Sc_0.4_O_3–*δ*_ was initially found to be phase pure by PXRD, subsequent NPD data revealed the presence of some Sc_2_O_3_, and hence the possible presence of BaSnO_3_ in the dried BaSn_0.6_Sc_0.4_O_3–*δ*_ sample. Indeed, very weak intensities, seen as shoulders on the main perovskite peaks, were visible in the NPD pattern (Fig. S1†) and the refined cell parameter obtained for the minor BaSnO_3_ component of 4.11457(17) Å showed good agreement with that of the dried BaSnO_3_ sample (*a* = 4.11588(2) Å). This was confirmed by the presence of the typical Sn(OSn)_6_ resonance at –679 ppm in the ^119^Sn NMR data.

On hydrothermal treatment of BaSn_0.6_Sc_0.4_O_3–*δ*_ with D_2_O, the minor Sc_2_O_3_ impurity was not observed in the NPD data, possibly indicating the complete solubility of the Sc_2_O_3_ into the perovskite structure is obtained under these conditions; a small signal characteristic of the local Sn environment present in BaSnO_3_ was still observed by ^119^Sn NMR (Fig. S2†). The significant difference in melting points of SnO_2_ (1630 °C) and Sc_2_O_3_ (2485 °C) reactants suggests that different cation diffusion rates are likely to be a contributing factor for the observed sample inhomogeneity across the BaSn_1–*x*_Sc_*x*_O_3–*δ*_ series. The solution based synthesis approaches utilised by Wang *et al.* ^10^ and Buannic *et al.* ^32^ in the preparation of BaSn_1–*x*_Y_*x*_O_3–*δ*_ (0.0 ≤ *x* ≤ 0.5) may be expected to help overcome this issue, although we note that the presence of undoped BaSnO_3_ was also reported for BaSn_0.9_Y_0.1_O_3–*δ*_.^32^

### 4.2. Deuteron site

**Fig. 12:**
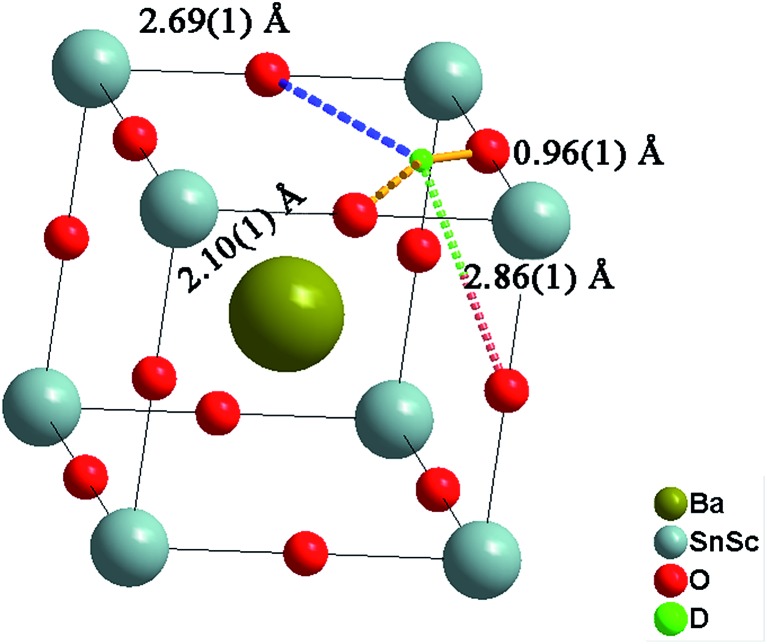
Illustration of the BaSn_0.6_Sc_0.4_O_2.6_(OD)_0.4_ unit cell showing the location of the deuteron site and its associated bond lengths to the nearest oxygen ions. The spheres represent in decreasing size Ba > Sn/Sc > O > D.

The deuteron site of the hydrothermally D_2_O treated BaSn_0.6_Sc_0.4_O_3–*δ*_ sample was successfully refined by Rietveld analysis. The deuteron atomic coordinates at the 24k site were *x* = 0.579(3) and *y* = 0.217(3) resulting in an average O–D bond distance of 0.96(1) Å, in good agreement with literature values.^21,42^ The local environment around a deuteron occupying the 24k site is illustrated in Fig. 12 and shows three O–D interatomic distances of relevance for the proton transfer step towards acceptor oxygen ions, the closest being at 2.11(1) Å. It is clear that the local proton configuration is highly anisotropic and these results are in good agreement with experimental studies of related, highly substituted, perovskites, BaZr_0.5_In_0.5_O_2.75_ (ref. 21) and BaSn_0.5_In_0.5_O_2.75_.^43^ The presence of a similar next nearest O–D interaction at ∼2.15 Å was also recently found for BaTi_0.5_In_0.5_O_2.53_(OD)_0.44_ from reverse Monte Carlo analysis of total scattering neutron diffraction data.^44^ These findings are in line with theoretical^45–47^ and experimental^48,49^ studies that revealed a clear tendency for protons to relax towards the dopant ions. The proximity of the second nearest oxygen atom indicates a tendency for enhanced hydrogen bonding interactions that will influence the proton diffusion. Whilst hydrogen bonding is expected to increase the likelihood of success for a proton transfer between neighbouring oxygen ions, the re-orientation step necessary for long range diffusion, involves breaking of the same H-bonds. It is therefore presently unclear what the full implications of the deuteron site are for migrating protons.

Both the D_2_O treated and the vacuum dried samples showed large and highly anisotropic ADPs for the oxygen site. This indicates significant static disorder of the oxygen ions as previously found^21^ and it is important to stress that the refined structural models will represent a long range, time averaged, picture.

The diffraction data for the deuterated BaSn_0.6_Sc_0.4_O_2.8_ sample revealed an increase in the cell parameter, *a*, compared to the as-prepared sample (Table 1). This lattice expansion is due to the filling of oxygen vacancies by larger hydroxyl (OD) groups and was similar in magnitude to that reported in related perovskites.^39,50^ The hydration process was also clearly reflected in the solid-state NMR data. The presence of a five-coordinate Sn and Sc peaks in the spectra of the dry BaSn_0.6_Sc_0.4_O_2.8_ sample confirmed the existence of oxygen vacancies 
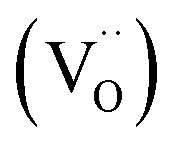
 and the loss of these signals upon hydrothermal D_2_O treatment confirmed their subsequent filling.

### 4.3. Local Sn and Sc environments

The fine structure observed for the contribution of each Y dopant cation in Sn(OSn)_6–*z*_(OY)_*z*_ (0 ≤ *z* ≤ 0.4) environment in the ^119^Sn NMR spectra of the related BaSn_1–*x*_Y_*x*_O_3–*δ*_ phases^31,32^ is not observed here for BaSn_0.6_Sc_0.4_O_3–*δ*_ (Fig. 4 and S2†). Additionally, a signal associated with Sn(OSn)_6_ environments persists for the highly substituted Sc sample whereas it is absent for BaSn_0.6_Y_0.4_O_2.8_ and BaSn_0.5_Y_0.5_O_2.75_.^32^ This suggests that there is greater disorder at the B-site and/or smaller changes in the specific local environments due to the much closer ionic radii of 6-fold coordinated Sn^4+^ (0.69 Å) and Sc^3+^ (0.745 Å) *vs.* 6-fold Y^3+^ (0.96 Å).^15^ More importantly, the presence of noticeable concentrations of Sn–O–Sn and Sc–O–Sc environments, as revealed by ^17^O NMR on ^17^O enriched BaSn_0.6_Sc_0.4_O_3–*δ*_, confirms the absence of significant Sn/Sc ordering; for strict ordering and *x* = 0.5, only the Sc–O–Sn environment should be present, analogous to the behaviour of BaSn_0.5_Y_0.5_O_2.75_ (Ba_2_SnYO_5.5_).^32^ For *x* < 0.5 this environment dominates, with lower concentrations of Sn–O–Sn environments being present, their concentration increasing with decreasing Sc content. For *x* = 0.1 and 0.2, the ratio of Sn(OSn)_6–*n*_(OSc)_*n*_ with *n* > 0 to Sn(OSn)_6_ sites is low but increases dramatically for *x* = 0.3 and 0.4, the amount of segregated BaSnO_3_ being minimum for the latter. There is a possibility that Sn, if hosting Sc in its vicinity, has a preference for hosting a high number of Sc, *i.e.* Sn(OSn)_6–*n*_(OSc)_*n*_ with *n* ≥ 3. In such case, the concentration of Sn(OSn)_6–*n*_(OSc)_*n*_ with *n* ≥ 3 would be small for *x* = 0.1 and 0.2 and, combined to a broad resonance (as seen for *x* = 0.3 and 0.4), would yield to a very weak signal. This hypothesis is corroborated by the recurring segregation of a non-negligible amount of BaSnO_3_, by the presence of a fair number of Sn–O–Sc bonds, and by the limited amount of Sc–O–Sc linkages as observed by ^17^O (Fig. 7) for *x* = 0.1 and 0.2. The greater size difference between Y^3+^ and Sn^4+^ drives a stronger tendency for ordering of the B-site cations that becomes nearly perfect with alternating Sn–O–Y–O–Sn linkages in BaSn_0.5_Y_0.5_O_2.75_ (Ba_2_SnYO_5.5_) as demonstrated by the existence a single main resonance at 259 ppm for Sn–O–Y moieties in the ^17^O NMR spectra.^32^ While some preferential cationic arrangement is possibly occurring in BaSn_1–*x*_Sc_*x*_O_3–*δ*_, it is not as predominant as in BaSn_1–*x*_Y_*x*_O_3–*δ*_.

The one-dimensional ^45^Sc NMR spectra of dry BaSn_0.8_Sc_0.2_O_3–*δ*_ (Fig. 5) reveals a clear signal for five coordinated Sc, whilst the ^119^Sn data show no signal of 5-coordinated Sn for the *x* = 0.1 and 0.2 samples. Taken together, this strongly implies that oxygen vacancies are preferentially found in between or near Sc cations at low doping levels (*x* ≤ 0.2). These findings agree with the results of Buannic *et al.*,^35^ Oikawa *et al.* ^36,51^ (for *x* ≤ 0.1) on the related BaZr_1–*x*_Sc_*x*_O_3–*δ*_ system that suggested a tendency for the amount of 5 coordinated Sc to increase with doping level for *x* ≤ 0.2. Interestingly, the one-dimensional ^45^Sc NMR spectra reveal that the ratio of Sc^V^ to Sc^VI^ decreases as the Sc content increases above *x* = 0.2. A notable difference between the BaSn_1–*x*_Sc_*x*_O_3–*δ*_ and BaZr_1–*x*_Sc_*x*_O_3–*δ*_ systems is the level of dopant solubility which reaches a maximum for BaZr_1–*x*_Sc_*x*_O_3–*δ*_ at *x* ≈ 0.2,^35^ whereas the scandium incorporation level reaches *x* ≈ 0.35 for the nominal *x* = 0.4 BaSn_1–*x*_Sc_*x*_O_3–*δ*_ sample based on the Sc site occupancy refined from neutron diffraction analysis for the dry material (Table 1). Buannic *et al.*,^35^ speculated earlier that the avoidance of energetically unfavourable Sc–O–Sc linkages, that are expected to become more numerous in systems where B-site ordering does not occur, may be a driving force for phase segregation into Sc_2_O_3_ and BaZr_1–*x*_Sc_*x*_O_3–*δ*_ with lower *x*. The present NMR findings for BaSn_0.6_Sc_0.4_O_3–*δ*_, showing coexistence of Sn(OSn)_6_ and SnO_5_ coordinations and low levels of ScO_5_ (in comparison to the *x* = 0.2 sample), therefore points towards a relative abundance of 
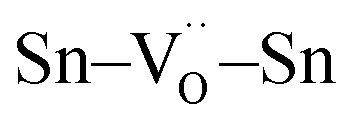
 local environments in comparison with a purely statistical cation and vacancy distribution. Possibly the Sn^4+^ ion is more flexible with respect to oxygen vacancies than Zr^4+^, and this plays a role in facilitating a relatively higher scandium incorporation into the perovskite matrix.

In summary, consideration of all the NMR data reveals an intricate picture in relation to the local B-site environments. The picture that emerges is nonetheless consistent with the formation of increasing levels of oxygen vacancies upon acceptor doping and the tendency for partial phase segregation, probably on nanometric-length scales, observed during the phase formation of the BaSn_1–*x*_Sc_*x*_O_3–*δ*_ series as discussed above.

### 4.4. Conductivity

The conductivity of BaSn_0.6_Sc_0.4_O_3–*δ*_ reveals a complex dependency on *p*O_2_ and *p*H_2_O. In dry, proton free, conditions the material reveals a major p-type contribution that is similar in magnitude to that reported recently for a related, highly acceptor doped, BaTi_0.5_Sc_0.5_O_3–*δ*_.^52^ The behaviour is also comparable to Ba_2_SnYO_5.5_,^9^ BaSn_1–*x*_Y_*x*_O_3–*δ*_ (ref. 12) and BaZr_0.8_Y_0.2_O_3–*δ*_ (ref. 53) for which hole conduction dominates at high *p*(O_2_) and high *T*. This enhanced conductivity can be rationalised through the partial filling of oxygen vacancies resulting in the formation of mobile electron holes as described in eqn (3) above.

Compared with the dry gas conditions, the conductivity under wet gas conditions (Ar or O_2_), and that obtained from the initial heating run in dry Ar on the pre-hydrated sample, were significantly higher in the intermediate temperature region of 150–650 °C (200–450 °C in wet O_2_) due to protons acting as the main charge carriers. Above 650 °C the conductivity obtained under wet and dry Ar were very similar as the material had probably dehydrated. This is characteristic of several related proton-conducting systems^9,54^ and reflects a transition to predominant oxide ion as protonic defects become unstable at higher *T*. Remarkably, at *T* > 450 °C under oxidizing conditions (Fig. 10), the conductivity is lower under wet gas compared to dry gas, although overall it still remains significantly higher than under wet Ar. This can be rationalised on the basis of the strong p-type character of the material that leads to a competition between holes and protons in wet oxidising conditions. A combination of the two reactions described by eqn (2) and (3) above would result in the consumption of holes in the presence of water vapour as per eqn (5) below.5
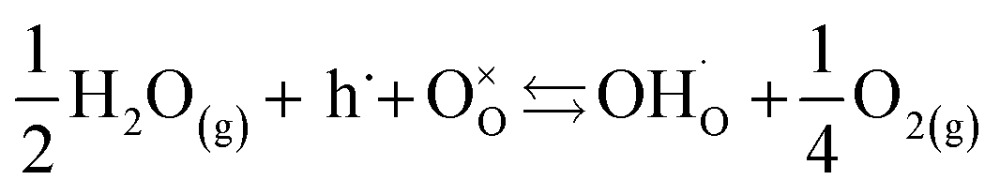



This consumption of holes depletes the number of available h˙ charge carriages and hence lowers the total conductivity at *T* > 450 °C to values below those of the dry oxygen condition. This kind of behaviour has been observed for BaZr_0.9–*x*_Pr_*x*_Gd_0.1_O_3–*δ*_ where highly mobile electron holes (h˙) were found to dominate conductivity even to very low temperatures and in wet conditions.^55^ It is clear that within the *T* range ∼400–600 °C, BaSn_0.6_Sc_0.4_O_3–*δ*_ displays significant mixed proton and electron conduction in wet oxygen, indicating potential suitability as a cathode material for PCFCs.

Table 3 lists a summary of obtained conductivity parameters for a number of acceptor doped BaSnO_3_ and BaTiO_3_ systems reported in the literature. For BaSn_0.6_Sc_0.4_O_3–*δ*_, it was possible to separate a bulk contribution for the initial heating run of the pre-hydrated sample at temperatures up to 350 °C. As apparent from the impedance data shown in Fig. 8 and the Arrhenius plot presented in Fig. 9, the total protonic conductivity is dominated by the highly resistive grain boundaries at relatively low temperatures. The bulk proton conduction in fact reaches a very high value of ∼2 × 10^–3^ S cm^–1^ at 350 °C (Fig. 9). The activation energy of bulk proton conductivity of BaSn_0.6_Sc_0.4_O_3–*δ*_, estimated from the heating cycle of the pre-hydrated sample in the *T* range 100 to 250 °C, was 0.40(1) eV. This shows reasonable agreement with the 0.52 eV reported for both of the more lightly Sc substituted BaSnO_3_ phases^10,56^ but is closer to the 0.38 eV and 0.34 eV reported for bulk proton conduction in BaSn_0.875_Y_0.125_O_3–*δ*_ (ref. 10) and BaSn_0.75_Y_0.25_O_3–*δ*_,^56^ respectively. The bulk activation energies reported by Wang *et al.* for BaSn_1–*x*_Y_*x*_O_3–*δ*_ (0.05 ≤ *x* ≤ 0.375)^12^ are approximately 0.1 eV lower than the 0.51 eV reported for bulk proton conductivity in Ba_2_YSnO_5.5_ (ref. 9) suggesting that the long range B-site ordering found in this phase is not beneficial to proton migration. Our present findings for BaSn_0.6_Sc_0.4_O_3–*δ*_ support this trend in as much as a low (0.40 eV) activation energy for bulk proton mobility is observed. Direct comparison of bulk activation energies is, however, not straightforward and values extracted from impedance data may also reflect partial contributions from, for example, defect formation enthalpies, dopant to proton trapping and the effects of grain boundaries. Therefore, although the trend from the data on acceptor doped BaSnO_3_ (Table 3) seemingly supports more facile bulk proton diffusion in disordered systems we avoid drawing wider conclusions in regards to the impact of B-site ordering on proton mobility in perovskites.

**Table 3:** Comparison of conductivity parameters with literature values of acceptor doped, BaSnO_3_ and BaTiO_3_ perovskite systems

Material	*E*_a_ protonic (total)/eV	*E*_a_ protonic (bulk)/eV	Total conductivity (protonic) in wet gas/S cm^–1^	Ref.
BaSn_0.9_In_0.1_O_3–*δ*_	—	0.54	1.3 × 10^–3^ (4% H_2_/96% N_2_ 500 °C)	11
BaSn_0.875_Sc_0.125_O_3–*δ*_	0.87	0.52	4 × 10^–4^(Ar, 500 °C)	10
BaSn_0.75_Sc_0.25_O_3–*δ*_	0.73	0.52	8 × 10^–4^ (Ar, 500 °C)	56
BaSn_0.6_Sc_0.4_O_3–*δ*_	0.7–0.67	0.40	1.07 × 10^–3^ (Ar, 600 °C)	This work
BaSn_0.9_Y_0.1_O_3–*δ*_	—	0.49	4 × 10^–5^ (4% H_2_/96% N_2_ 500 °C)	13
BaSn_0.875_Y_0.125_O_3–*δ*_	0.72	0.38	2.3 × 10^–4^ (Ar, 500 °C)	10
BaSn_0.75_Y_0.25_O_3–*δ*_	0.66	0.34	7 × 10^–4^ (Ar, 500 °C)	12
BaSn_0.5_Y_0.5_O_2.75_ (Ba_2_YSnO_5.5_ double perovskite structure)	—	0.51	1.3 × 10^–3^ (bulk) (N_2_, 600–400 °C)	9
BaTi_0.8_Sc_0.2_O_3–*δ*_ (6 H hexagonal structure)	0.80	0.77	1 × 10^–5^ (Ar, 400 °C)	58
BaTi_0.5_Sc_0.5_O_3–*δ*_	0.46	0.22	2.89 × 10^–4^ (Ar, 550 °C)	52
BaTi_0.3_Sc_0.7_O_3–*δ*_	0.48	—	2 × 10^–3^ (Ar, 600 °C)	58
BaTi_0.5_In_0.5_O_3–*δ*_	0.48	—	2.1 × 10^–4^ (Ar, 500 °C)	59
BaTi_0.2_In_0.8_O_3–*δ*_	—	0.42	1.1 × 10^–3^ (450 –600 °C)	57

The activation energies for the total protonic conduction of BaSn_0.6_Sc_0.4_O_3–*δ*_ lie in the range 0.67–0.70 eV in wet Ar which is lower than the 0.87 eV recently reported for BaSn_0.875_Sc_0.125_O_3–*δ*_,^10^ and closer to the 0.73 eV obtained for BaSn_0.75_Sc_0.25_O_3–*δ*_.^56^ The total proton conductivity of 1.07 × 10^–3^ S cm^–1^ obtained for BaSn_0.6_Sc_0.4_O_3–*δ*_ in wet Ar at 600 °C is similar to that of BaIn_0.8_Ti_0.2_O_2.6_ (ref. 57) and BaTi_0.3_Sc_0.7_O_3–*δ*_,^58^ and is significantly higher than that reported previously for BaSn_1–*x*_Sc_*x*_O_3–*δ*_ with lower scandium contents.^10,11^ This behaviour probably reflects the greater proton concentration in the more highly doped system. A trend of increasing proton conduction with increasing dopant concentration might be emerging from Table 3. Ultimately, however, it is the proton mobility, and understanding how it is influenced by factors such as the level of B-site cation ordering and the chemical nature of the ions, that is critical in order to obtain new materials with significantly enhanced proton conductivity.

## 5. Conclusions

Scandium substitution of the tin site within BaSnO_3_ has been achieved by solid-state synthesis. Some degree of phase segregation was observed in the dry materials but it has largely disappeared in the BaSn_0.6_Sc_0.4_O_2.8_ sample after D_2_O treatment. Analysis of X-ray and neutron diffraction data has indicated an average cubic symmetry of space group *Pm*3*m* and the deuteron position was successfully located at the 24k site (0.579(3), 0.217(3), 0) from Rietveld analysis. ^119^Sn solid-state NMR revealed a series of local tin environments consistent with 6 and 5 coordinate Sn environments and BaSnO_3_ impurities. The resonance from the 6-coordinate site is broad indicating a wide range of Sn environments differing in the number of Sn and Sc cation in the 1^st^ B-site cation coordination shell. This behaviour is very different to the structure of BaSn_1–*x*_Y_*x*_O_3–*δ*_ with a high concentration of yttrium, in which Y–O–Sn ordering occurs. The five-coordinated Sn is observed at high Sc doping levels (*x* ≥ 0.4), confirming the presence of oxygen vacancies nearby tin. Conversely, the ^45^Sc NMR data showed the existence of intense peaks for five-coordinated Sc, the relative fraction of five to six-coordinated Sc increasing with decreasing Sc content, suggesting preferential trapping of oxygen vacancies in between or near Sc cations at lower Sc concentrations. For all compositions, the five coordinated Sc and Sn environments vanished after hydration as OH groups filled the available oxygen vacancies.

BaSn_0.6_Sc_0.4_O_3–*δ*_ was found to be predominantly a p-type conductor under oxidizing atmospheres with proton conduction dominating at lower temperatures. The competition between holes and protons results in a suppression of the conductivity at *T* > 450 °C in wet oxidizing conditions in comparison to dry oxygen. This mixed ionic and electron hole conduction means that the material could be utilized in gas separation membrane applications or cathodes of proton conducting fuel cells. The current study showed that highly scandium substituted BaSnO_3_ supports very high bulk proton conductivity, comparable to that reported for Y-doped BaZrO_3_ and the double perovskite Ba_2_YSnO_5.5_. It is suggested that further work on the material should focus on the growth of large grained samples in order to reduce grain boundary resistance, and investigate the chemical stability of the material under CO_2_ atmospheres. Given the present findings, solution synthesis routes aiming to create a more homogeneous mixing of the B-site ions at the atomic level are also of potential interest.

## Authors' contribution

I. A., S. G. E., S. T. N. conceptualized and planned the project, F. G. K., I. A. synthesized the samples. F. G. K. did EIS experiments and EIS data analysed with C. S. K., F. G. K., I. A., S. T. N., S. H., S. G. E. performed neutron data collection and structural analysis. L.B. prepared the ^17^O enriched materials. L. B., I. H., Z. G. and F. B. carried out the NMR experiments. L. B., F. B. and C. P. G. performed analysis of the NMR data. The manuscript was written with contribution from all co-authors.

## References

[cit1] Jiang S. P., Zhen Y. D. (2008). Solid State Ionics.

[cit2] Kreuer K. D. (2003). Annu. Rev. Mater. Res..

[cit3] Yamazaki Y., Hernandez-Sanchez R., Haile S. M. (2009). Chem. Mater..

[cit4] Fabbri E., Pergolesi D., Traversa E. (2010). Chem. Soc. Rev..

[cit5] Iwahara H., Esaka T., Uchida H., Maeda N. (1981). Solid State Ionics.

[cit6] Slade R. C. T., Flint S. D., Singh N. (1995). Solid State Ionics.

[cit7] Manthiram A., Kuo J. F., Goodenough J. B. (1993). Solid State Ionics.

[cit8] Schober T. (1998). Solid State Ionics.

[cit9] Murugaraj P., Kreuer K. D., He T., Schober T., Maier J. (1997). Solid State Ionics.

[cit10] Wang Y., Chesnaud A., Bevillon E., Yang J., Dezanneau G. (2011). Int. J. Hydrogen Energy.

[cit11] Wang Y., Chesnaud A., Bévillon E., Xiong J., Yang J. (2013). J. Alloys Compd..

[cit12] Wang Y. Z., Chesnaud A., Bevillon E., Dezanneau G. (2012). Solid State Ionics.

[cit13] Li L. P., Nino J. C. (2013). Int. J. Hydrogen Energy.

[cit14] Bévillon É., Hermet J., Dezanneau G., Geneste G. (2014). J. Mater. Chem. A.

[cit15] Shannon R. D. (1976). Acta Crystallogr., Sect. A: Cryst. Phys., Diffr., Theor. Gen. Crystallogr..

[cit16] Hull S., Smith R. I., David W. I. F., Hannon A. C., Mayers J., Cywinski R. (1992). Phys. B.

[cit17] Larson R. B. V. D. A. C., Los Alamos National Laboratory Report, LAUR 86-748.

[cit18] Toby B. H. (2001). J. Appl. Crystallogr..

[cit19] Rietveld H. M. (1969). J. Appl. Crystallogr..

[cit20] McCusker L. B., Von Dreele R. B., Cox D. E., Louer D., Scardi P. (1999). J. Appl. Crystallogr..

[cit21] Ahmed I., Knee C. S., Karlsson M., Eriksson S. G., Henry P. F., Matic A., Engberg D., Börjesson L. (2008). J. Alloys Compd..

[cit22] Gan Z., Gor'kov P. L., Brey W. W., Sideris P. J., Grey C. P. (2009). J. Magn. Reson..

[cit23] Frydman L., Harwood J. S. (1995). J. Am. Chem. Soc..

[cit24] Medek A., Harwood J. S., Frydman L. (1995). J. Am. Chem. Soc..

[cit25] Amoureux J.-P. and Pruski M., in eMagRes, John Wiley & Sons, Ltd, 2007, 10.1002/9780470034590.emrstm0319.pub2.

[cit26] Gan Z., Kwak H. T. (2004). J. Magn. Reson..

[cit27] Amoureux J.-P., Fernandez C., Steuernagel S. (1996). J. Magn. Reson., Ser. A.

[cit28] van Beek J. D. (2007). J. Magn. Reson..

[cit29] Dusek M., Petricek V., Palatinus L. (2006). Acta Crystallogr., Sect. A: Found. Crystallogr..

[cit30] Cerda J., Arbiol J., Dezanneau G., Diaz R., Morante J. R. (2002). Sens. Actuators, B.

[cit31] Clayden N. J., Dobson C. M., Fern A. (1989). J. Chem. Soc., Dalton Trans..

[cit32] Buannic L., Blanc F., Middlemiss D. S., Grey C. P. (2012). J. Am. Chem. Soc..

[cit33] MacKenzie K. J. D. and Smith M. E., Multinuclear Solid-State Nmr of Inorganic Materials, Elsevier, 2002.

[cit34] Kim N., Hsieh C.-H., Stebbins J. F. (2006). Chem. Mater..

[cit35] Buannic L., Blanc F., Hung I., Gan Z. H., Grey C. P. (2010). J. Mater. Chem..

[cit36] Oikawa I., Takamura H. (2015). Chem. Mater..

[cit37] Kim N., Grey C. P. (2002). Science.

[cit38] Ashbrook S. E., Smith M. E. (2006). Chem. Soc. Rev..

[cit39] Ahmed I., Eriksson S. G., Ahlberg E., Knee C. S., Berastegui P., Johansson L. G., Rundlof H., Karlsson M., Matic A., Borjesson L., Engberg D. (2006). Solid State Ionics.

[cit40] Roth R. S. (1957). J. Res. Natl. Bur. Stand. (U. S.).

[cit41] Hiraiwa C., Han D., Kuramitsu A., Kuwabara A., Takeuchi H., Majima M., Uda T. (2013). J. Am. Ceram. Soc..

[cit42] Kendrick E., Knight K. S., Islam M. S., Slater P. R. (2007). Solid State Ionics.

[cit43] Ito T., Nagasaki T., Iwasaki K., Yoshino M., Matsui T., Igawa N., Ishii Y. (2007). Solid State Ionics.

[cit44] Norberg S. T., Rahman S. M., Hull S., Knee C. S., Eriksson S. G. (2013). J. Phys.: Condens. Matter.

[cit45] Karlsson M., Björketun M. E., Sundell P. G., Matic A., Wahnström G., Engberg D., Börjesson L., Ahmed I., Eriksson S., Berastegui P. (2005). Phys. Rev. B: Condens. Matter Mater. Phys..

[cit46] Shi C., Morinaga M. (2006). J. Comput. Chem..

[cit47] Stokes S. J., Islam M. S. (2010). J. Mater. Chem..

[cit48] Yamazaki Y., Blanc F., Okuyama Y., Buannic L., Lucio-Vega J. C., Grey C. P., Haile S. M. (2013). Nat. Mater..

[cit49] Blanc F., Sperrin L., Lee D., Dervisoglu R., Yamazaki Y., Haile S. M., De Paepe G., Grey C. P. (2014). J. Phys. Chem. Lett..

[cit50] Sosnowska I., Przeniosło R., Schäfer W., Kockelmann W., Hempelmann R., Wysocki K. (2001). J. Alloys Compd..

[cit51] Oikawa I., Ando M., Noda Y., Amezawa K., Kiyono H., Shimizu T., Tansho M., Maekawa H. (2011). Solid State Ionics.

[cit52] Rahman S. M. H., Ahmed I., Haugsrud R., Eriksson S. G., Knee C. S. (2014). Solid State Ionics.

[cit53] Nomura K., Kageyama H. (2007). Solid State Ionics.

[cit54] Ahmed I., Eriksson S. G., Ahlberg E., Knee C. S., Gotlind H., Johansson L. G., Karlsson M., Matic A., Borjesson L. (2007). Solid State Ionics.

[cit55] Magrasó A., Frontera C., Gunnæs A. E., Tarancón A., Marrero-López D., Norby T., Haugsrud R. (2011). J. Power Sources.

[cit56] Wang Y., PhD Doctorate, Ecole Centrale Paris, Chine, 2009.

[cit57] Quarez E., Noirault S., Caldes M. T., Joubert O. (2010). J. Power Sources.

[cit58] Rahman S. M., Norberg S. T., Knee C. S., Biendicho J. J., Hull S., Eriksson S. G. (2014). Dalton Trans..

[cit59] Rahman S. M. H., Knee C. S., Ahmed I., Eriksson S. G., Haugsrud R. (2012). Int. J. Hydrogen Energy.

