# Supplementary material for: Crystal structure and proton conductivity of BaSn0.6Sc0.4O3–δ: insights from neutron powder diffraction and solid-state NMR spectroscopy
Source: J Mater Chem A Mater. 2016 Mar 16;4(14):5088–101. doi: 10.1039/c5ta09744d (PMC4894074; doi:10.1039/c5ta09744d)
Supplement: Supplementary file 1 [file TA-004-C5TA09744D-s001.pdf]

Electronic Supplementary Material (ESI) for Journal of Materials Chemistry A.
This journal is © The Royal Society of Chemistry 2016

Supplementary Information:

Crystal structure and proton conductivity of $BaSn_{0.6}Sc_{0.4}O_{3-\delta}$: Insights from neutron powder diffraction and solid state NMR spectroscopy

Francis G. Kinyanjui, [1,*] Stefan T. Norberg, [1] Christopher S. Knee, [1] Istaq Ahmed, [1] Stephen Hull, [2] Lucienne Buannic, [3] Ivan Hung, [4] Zhehong Gan, [4] Frédéric Blanc, [5, 6] Clare P. Grey, [3, 5] Sten G. Eriksson [1]

[1] Department of Chemical and Biological Engineering, Chalmers University of Technology, SE-412 96 Gothenburg, Sweden

[2] The ISIS Facility, STFC Rutherford Appleton Laboratory, Didcot, Oxfordshire, OX11 0QX, United Kingdom

[3] Department of Chemistry, State University of New York, Stony Brook, NY 11790-3400, USA

[4] Center of Interdisciplinary Magnetic Resonance, National High Magnetic Field Laboratory, 1800 East Paul Dirac Drive, Tallahassee, Florida 32310, United States

[5] Department of Chemistry, University of Cambridge, Lensfield Road, Cambridge, CB2 1EW, United Kingdom

[6] Department of Chemistry and Stephenson Institute for Renewable Energy, University of Liverpool, Crown Street, Liverpool, L69 7ZD, United Kingdom

* Corresponding author: Francis G. Kinyanjui

Present address:

Department of Materials,
University of Oxford,
OX1 3PH Oxford,
United Kingdom,
E-mail: francis.kinyanjui@materials.ox.ac.uk
Phone: +44 1865 612765

Additional Figures

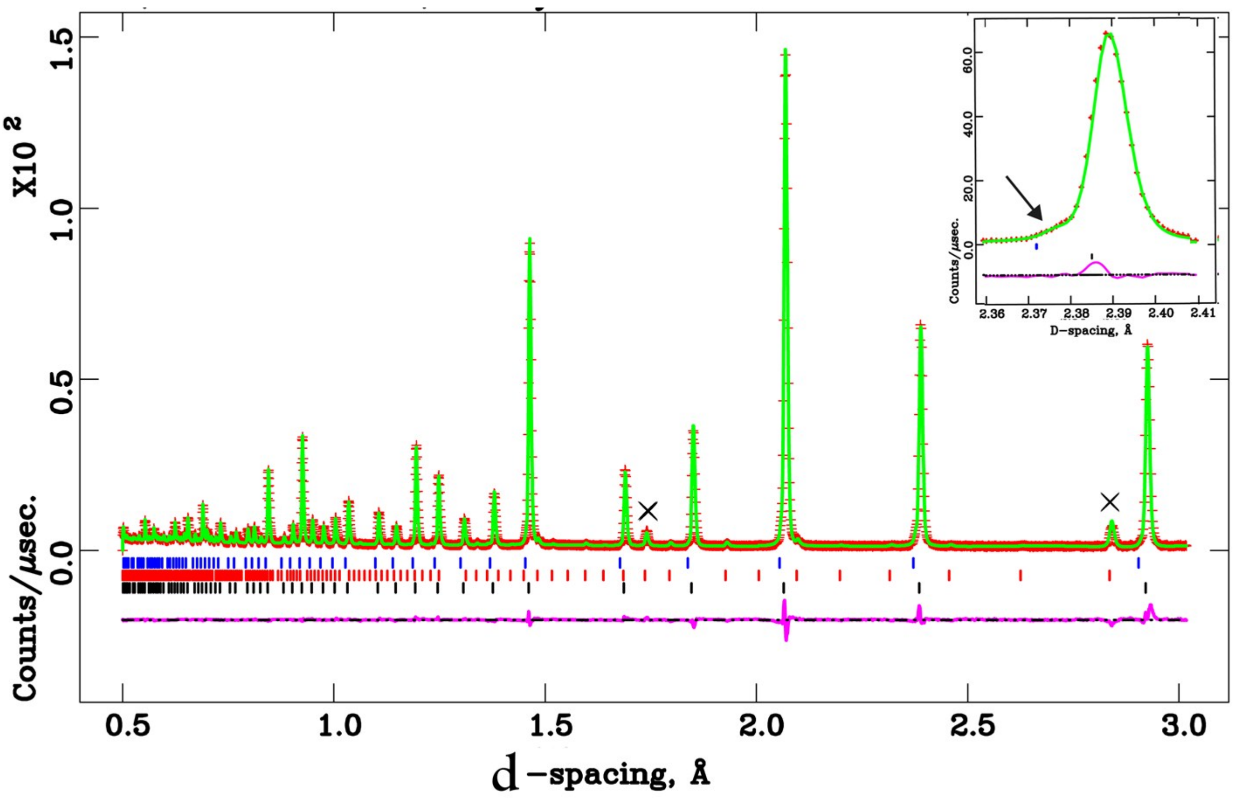

Figure S1 Rietveld fit to the data for dried $BaSn_{0.6}Sc_{0.4}O_{3-\delta}$ sample showing the presence of three modelled phases, $BaSnO_3$ (blue bars) $BaSn_{0.6}Sc_{0.4}O_{3-\delta}$ (black bars) and Sc_2O_3 (red bars, black crosses). The insert shows a zoomed in section with an arrow indicating the peak shoulder due to $BaSnO_3$. Both the observed (red crosses) and calculated (continuous green line) profiles are plotted and the position of the reflections is marked with vertical bars. The difference curve lies at the bottom in purple.

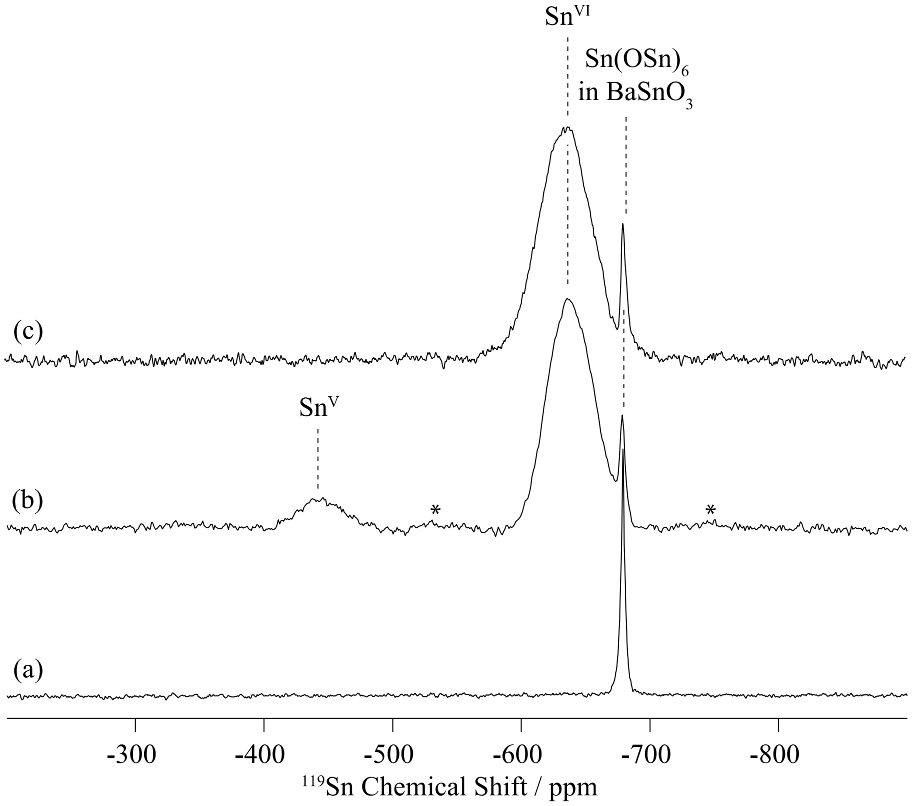

Figure S2 ^{119}Sn MAS NMR single pulse spectra of (a) dry $BaSnO_3$, (b) dry $BaSn_{0.6}Sc_{0.4}O_{3-\delta}$ and (c) deuterated $BaSn_{0.6}Sc_{0.4}O_{3-\delta}$ obtained at 11.7 T and under a MAS frequency of 20 kHz. Sn^{VI} and Sn^{V} denote six and five coordinated tin environments. Asterisks (*) denote spinning side bands.

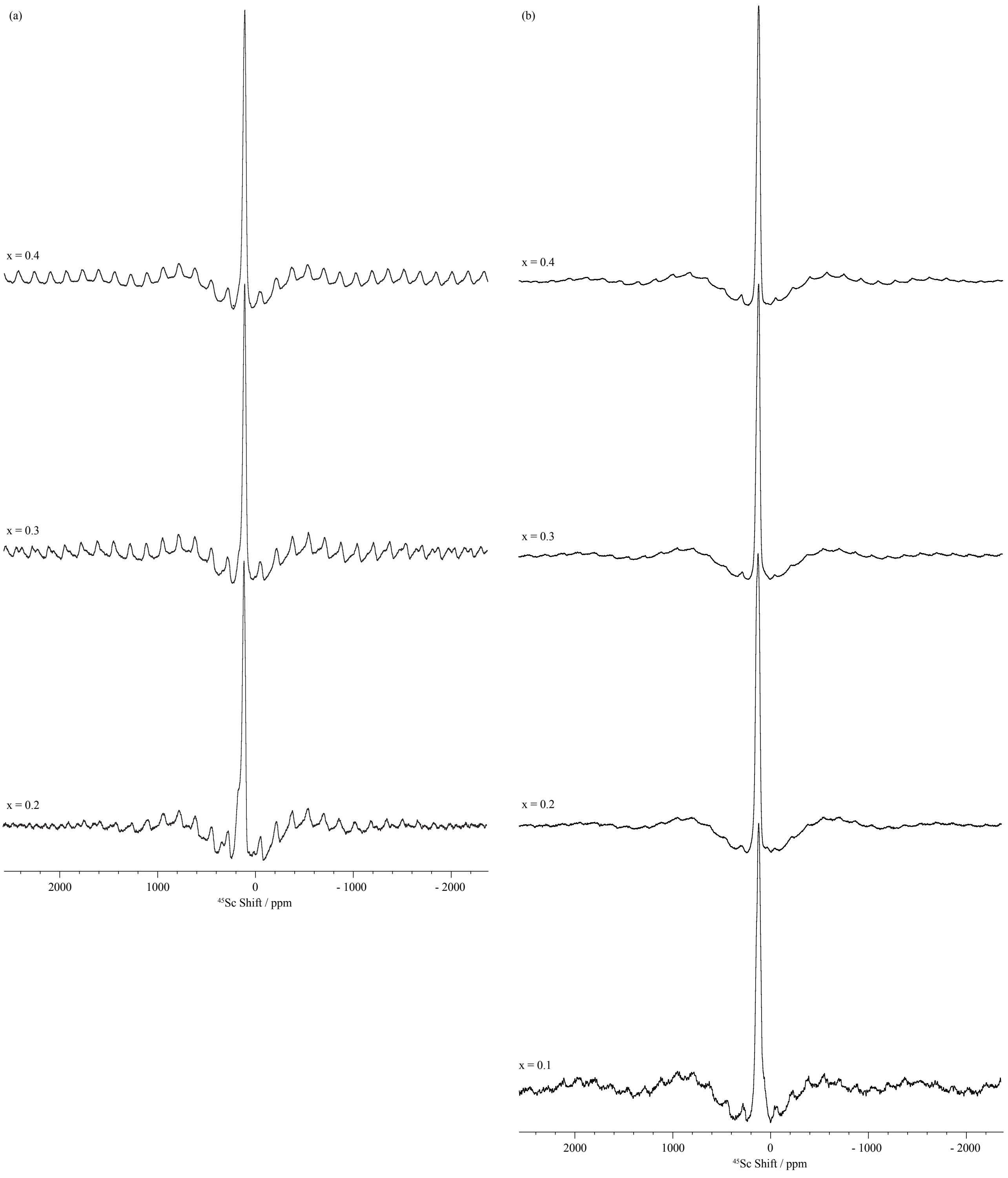

Figure S3 ^{45}Sc MAS NMR spectra of (a) dry $BaSn_{1-x}Sc_xO_{3-\delta}$ and (b) deuterated $BaSn_{1-x}Sc_xO_{3-\delta}$ as a function of Sc doping level x. The spectra were obtained at 19.6 T and under MAS frequency of 33.333 kHz. The central transition region is found between 300 and - 50 ppm (see Figure S4). The ^{45}Sc MAS NMR spectra of dry $BaSn_{0.9}Sc_{0.1}O_{3-\delta}$ was not recorded.

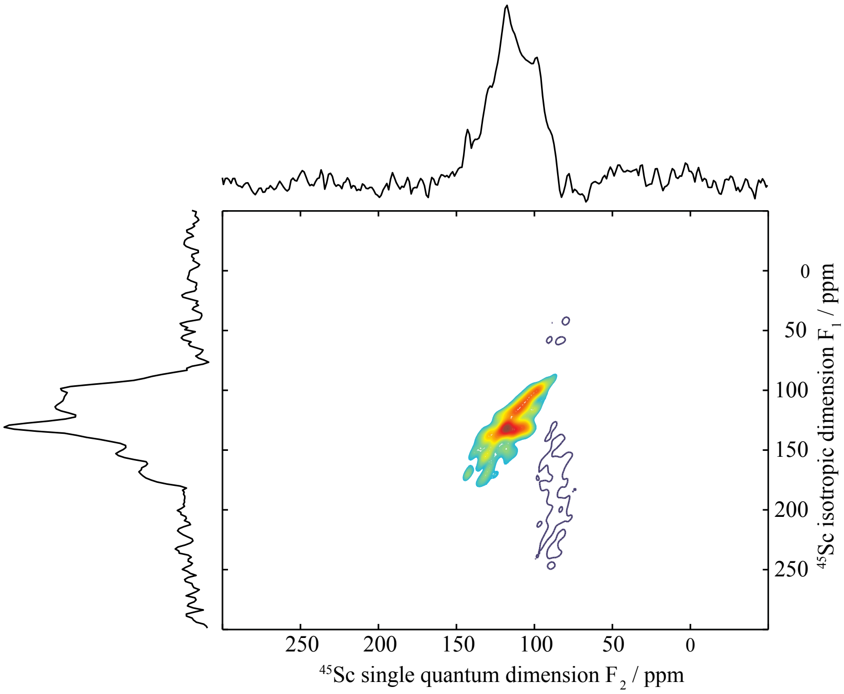

Figure S4 Two-dimensional sheared triple-quantum ^{45}Sc MAS spectra of deuterated $BaSn_{0.9}Sc_{0.1}O_{3-\delta}$ obtained at 19.6 T and under MAS frequency of 33.333 kHz. 9600 transients were accumulated for each of the 16 t_1 increments with recycle delays of 0.4 s. Top: anisotropic skyline projection (the ^{45}Sc MAS NMR single pulse spectra are given in Figure S3). Left: isotropic skyline projection of the 3Q MAS spectra.

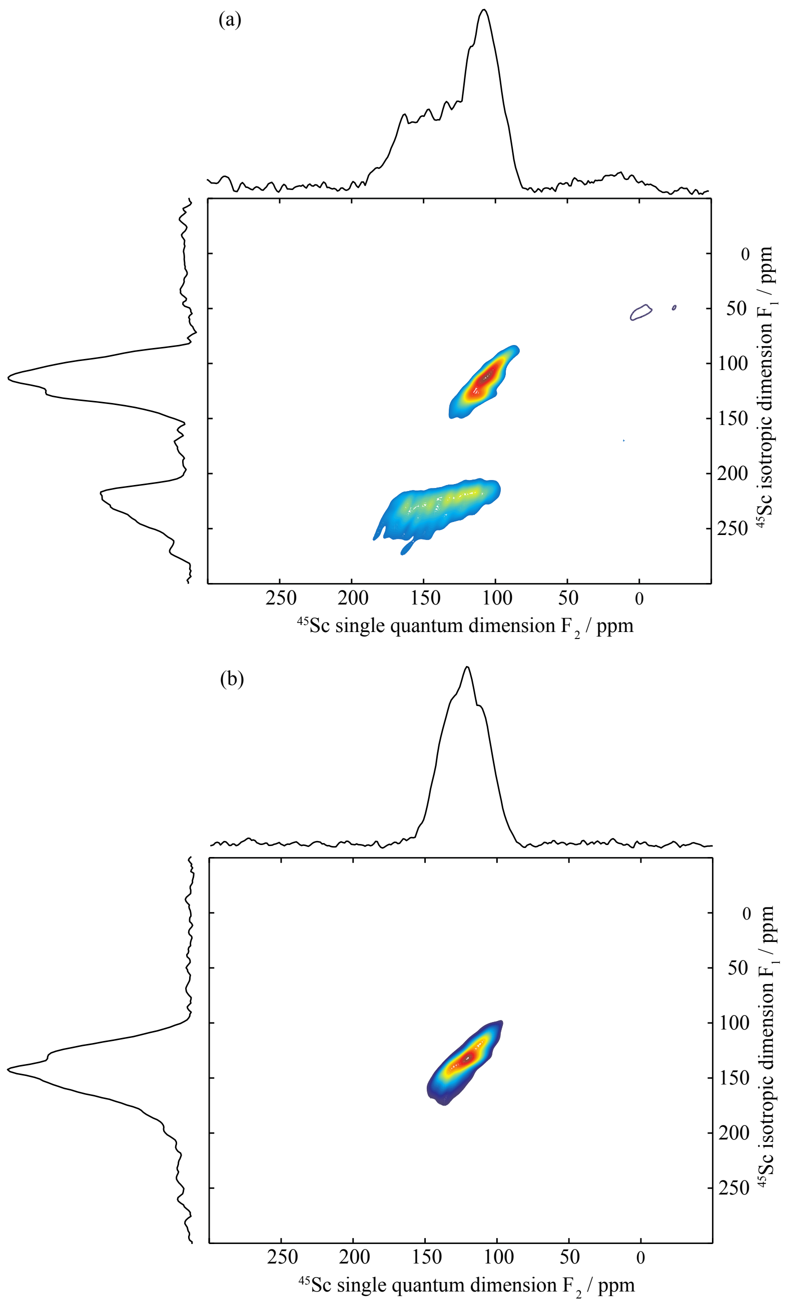

Figure S5 Two-dimensional sheared triple-quantum ^{45}Sc MAS spectra of (a) dry $BaSn_{0.8}Sc_{0.2}O_{3-\delta}$ and (b) deuterated $BaSn_{0.8}Sc_{0.2}O_{3-\delta}$ obtained at 19.6 T and under MAS frequency of 33.333 kHz. 9600 transients were accumulated for each of the 24 (for (a)) and 16 (for (b)) t_1 increments with recycle delays of 0.4 s. Top: anisotropic skyline projection (the ^{45}Sc MAS NMR single pulse spectra are given in Figures S3). Left: isotropic skyline projection of the 3Q MAS spectra.

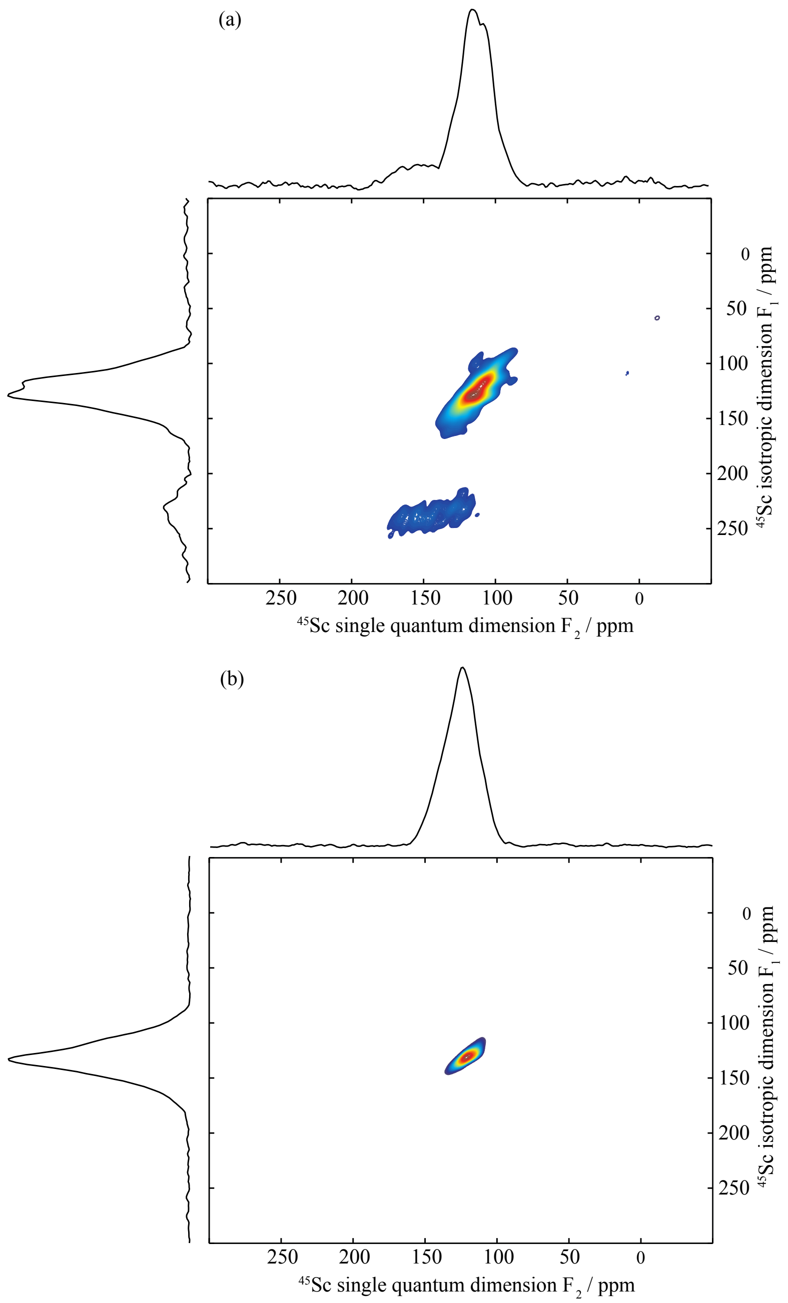

Figure S6 Two-dimensional sheared triple-quantum ^{45}Sc MAS spectra of (a) dry $BaSn_{0.7}Sc_{0.3}O_{3-\delta}$ and (b) deuterated $BaSn_{0.7}Sc_{0.3}O_{3-\delta}$ obtained at 19.6 T and under MAS frequency of 33.333 kHz. 9600 transients were accumulated for each of the 24 (for (a)) and 16 (for (b)) t_1 increments with recycle delays of 0.4 s. Top: anisotropic skyline projection (the ^{45}Sc MAS NMR single pulse spectra are given in Figures S3-S4). Left: isotropic skyline projection of the 3Q MAS spectra.

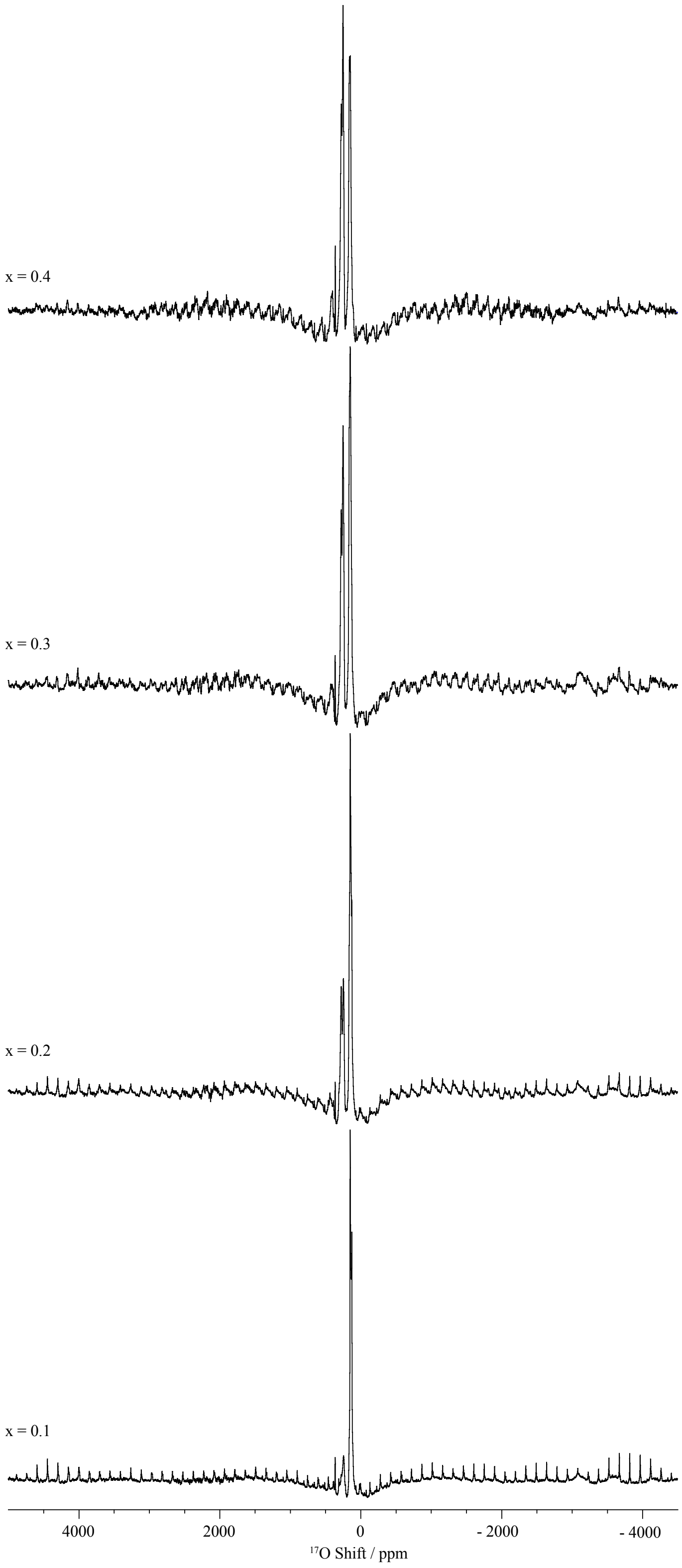

Figure S7 ^{17}O MAS NMR single pulse spectra of ^{17}O enriched $BaSn_{1-x}Sc_xO_{3-\delta}$ as a function of the Sc doping level x. The spectra were obtained at 17.6 T and under MAS frequency of 15 kHz. The central transition region is found between 600 and - 200 ppm (see Figure S5).

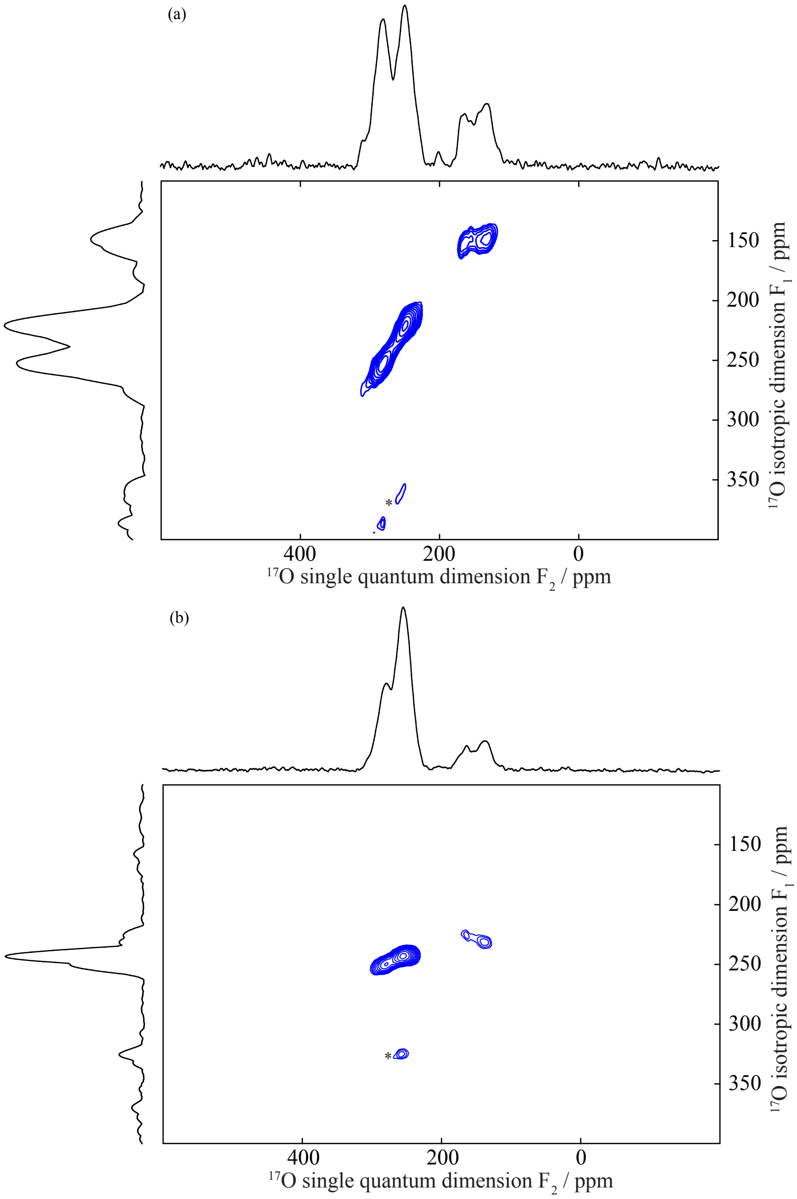

Figure S8 Two-dimensional sheared triple-quantum ^{17}O MAS spectra of ^{17}O enriched (a) $BaSn_{0.8}Sc_{0.2}O_{3-\delta}$ and (b) $BaSn_{0.6}Sc_{0.4}O_{3-\delta}$ obtained at 17.6 T and under MAS frequency of 15 kHz. 1200 transients were accumulated for each of the 38 t_1 increments used with recycle delays of 3 s. Top: anisotropic skyline projection (the ^{17}O MAS NMR single pulse spectra are given in Figures S8-S9). Left: isotropic skyline projection of the 3Q MAS spectra. The asterisks (*) denote the spinning side bands.
